# Supplementary material for: Mechanical overtone frequency combs
Source: Nat Commun. 2023 Mar 16;14:1458. doi: 10.1038/s41467-023-36953-8 (PMC10020424; doi:10.1038/s41467-023-36953-8)
Supplement: Supplementary file 1 — Supplementary Information [file 41467_2023_36953_MOESM1_ESM.pdf]

Supplementary Information: Mechanical overtone frequency combs

Matthijs H. J. de Jong,[1,2] Adarsh Ganesan,[3,4] Andrea Cupertino,[1] Simon Gröblacher,[2] and Richard A. Norte[1,2]

[1] *Department of Precision and Microsystems Engineering, Delft University of Technology, Mekelweg 2, 2628CD Delft, The Netherlands*

[2] *Kavli Institute of Nanoscience, Department of Quantum Nanoscience, Delft University of Technology, Lorentzweg 1, 2628CJ Delft, The Netherlands*

[3] *Ahmedabad University, Ahmedabad, Gujarat 380009, India*

[4] *National Institute of Standards and Technology, Gaithersburg, Maryland 20899, USA*

(Dated: January 4, 2023)

I. SUPPLEMENTARY INFORMATION

This document contains the supplementary information.

A. Mechanical overtones

The motion of a mechanical resonator can typically be described by considering a linear superposition of the normal modes of the structure. In some structures, the normal modes are related by integer multiples of some frequency, and they are said to be *harmonic*. This is the case for a model string (Fig. S1**a,b**) and a desirable property for many musical instruments. However for most of the resonators in this work, the normal modes are not related by integer multiples, thus the spectrum is *anharmonic*. In Fig. S1**c**, we have simulated and plotted the first six eigenmodes of the trampoline membrane resonator of the main text, and plotted their frequencies in Fig. S1**b**.

In this work, we introduce an optics-based non-linearity to the equation of motion, which affects the description of the resonator motion in terms of the normal modes given by its structure. Briefly summarized, we consider only the lowest frequency normal mode given by the mechanical structure, as a simple harmonic oscillator,

$$\ddot{x} + \gamma\dot{x} + \omega_0^2 x = 0. \tag{S1}$$

The solutions for this equation are well-known, $x \propto e^{i\omega_0 t}$, which oscillates at the frequency ω_0 of the normal mode. By introducing the gradient force term, $\propto \sin(x)$ (non-linear), we get terms in our solution that oscillate at integer multiples of ω_0, without being related to the (other) normal modes of the mechanical resonator. To distinguish them from the other normal modes, we will refer to the components at $n\omega_0$ (n integer) as **overtones**. Others have detected these components of the mechanical motion before, and refer to them in a similar manner [1–3]. They ascribe the origin of these overtones to be mechanical in nature, while we propose an optical origin.

1. *Small displacement*

To expand the description of these overtones, we consider the equation of motion as Eq. 1 in the main text, where we have added a resonant drive term F_d and absorbed the effective mass m_eff into F_o and F_d for convenience. The shorthand $\Lambda = 4\pi/\lambda$ is similarly used. We get

$$\ddot{x} + \gamma\dot{x} + \omega_0^2 x = F_\mathrm{o}\sin\left(\Lambda(x - x_\mathrm{off})\right) + F_\mathrm{d}e^{i\omega_0 t}. \tag{S2}$$

The periodic part of the optical potential is of the form $E \propto \sin\left(\frac{2\pi}{\lambda}(x - x_\mathrm{off})\right)$. The gradient of the optical intensity, $\nabla E^2 \propto \sin\left(\Lambda(x - x_\mathrm{off})\right)$ after some algebra and absorbing the relevant constants into F_o. The constant offset between the potentials, x_off, can be taken out of the sine by

$$\sin\left(\Lambda(x - x_\mathrm{off})\right) = \sin\left(\Lambda x\right) s_x + \cos\left(\Lambda x\right) c_x, \tag{S3}$$

with $s_x = \cos(\Lambda x_\mathrm{off})$ and $c_x = \sin(\Lambda x_\mathrm{off})$. For a small displacement x, we can use the Taylor series and truncate the higher order terms, so $s_x \sin(x) + c_x \cos(x) \simeq c_x + s_x x - \frac{c_x}{2}x^2$. The equation of motion then becomes

$$\ddot{x} + \gamma\dot{x} + \omega_0^2 x = F_\mathrm{o}\left(c_x + s_x\Lambda x - \frac{c_x}{2}\Lambda^2 x^2\right) + F_\mathrm{d}e^{i\omega_0 t}. \tag{S4}$$

This equation admits solutions of the form

$$x = \sum_{n=0}^{\infty} A_n e^{in\omega_0 t}, \tag{S5}$$

which are the integer multiples of our original frequency ω_0; the overtones. The term $n = 0$ corresponds to a static position offset from the zero of the mechanical potential. Substituting the solution Eq. (S5) into Eq. (S4) and gathering all terms by their frequency ($e^{in\omega_0 t}$ for every n separately) allows us to extract the amplitudes of the individual overtones in terms of the parameters of our system. We get

$$\begin{aligned}
A_0 &\simeq c_x F_\mathrm{o}/\omega_0^2 \\
A_1 &= \frac{F_\mathrm{d}}{i\gamma\omega_0 - \Lambda F_\mathrm{o} s_x + F_\mathrm{o}\frac{c_x}{2}\Lambda^2 A_0}, \\
A_2 &= \frac{F_\mathrm{o}\frac{c_x}{2}\Lambda^2 A_1^2}{3\omega_0^2 - 2i\gamma\omega_0 + F_\mathrm{o}s_x\Lambda - F_\mathrm{o}\frac{c_x}{2}\Lambda^2 A_0}, \\
A_n &= \frac{F_\mathrm{o}\Lambda \sum_{j,k=0}^{\infty} A_j A_k}{(n^2-1)\omega_0^2 - i\gamma n\omega_0 + F_\mathrm{o}s_x\Lambda - F_\mathrm{o}\frac{c_x}{2}\Lambda^2 A_0},
\end{aligned} \tag{S6}$$

where the summation contains only the terms where $j + k = n$. This sequence of overtone amplitudes is monotonically decreasing (see Fig. S2): Every $A_n < A_{n-1}$. In the small-displacement case, the overtones will be negligible compared to A_1.

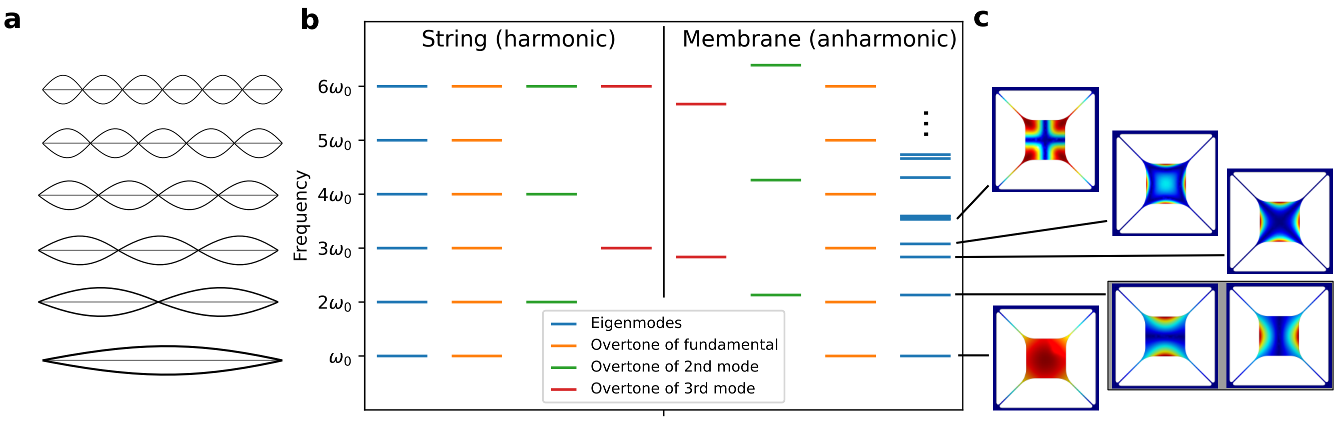

FIG. S1. **Mechanical overtones.** **a**: Resonator modes of a string. **b**: Frequency diagram of eigenmodes of a harmonic string and the anharmonic SiN trampoline membranes used in this work. **c**: SiN trampoline modes, grey box denotes degenerate modes. The color scale denotes normalized absolute displacement, from minimum (blue) to maximum (red).

2. *Large displacement*

If instead the displacement is not small, we can still use the same method. We need to keep all the terms of the Taylor expansion,

$$\sin(x) = \sum_{n=0}^{\infty} \frac{(-1)^n x^{2n+1}}{(2n+1)!}, \quad \cos(x) = \sum_{n=0}^{\infty} \frac{(-1)^n x^{2n}}{(2n)!}. \tag{S7}$$

We can use the same ansatz of Eq. (S5), and extract the amplitudes by collecting all terms of the same frequency. For $n = 0$, the solution converges to

$$A_0 \simeq c_x F_o/\omega_0^2, \tag{S8}$$

as the contribution from the higher order terms of the expansion scale with $1/\Lambda^n$. Similarly, the denominator for $n > 0$ can be truncated to obtain

$$\begin{aligned} A_1 &= \frac{F_d}{i\gamma\omega_0 - \Lambda F_o s_x + F_o \frac{c_x}{2}\Lambda^2 A_0} \\ A_n &= \frac{F_o \sum_j \left(s_x s_j \sum\limits_{k,\ell,\ldots=0}^{\infty} A^j_{k,\ell,\ldots} + c_x c_j \sum\limits_{k,\ell,\ldots=0}^{\infty} A^j_{k,\ell,\ldots} \right)}{(n^2-1)\omega_0^2 - i\gamma n\omega_0 + \Lambda F_o s_x - F_o \frac{c_x}{2}\Lambda^2 A_0}, \end{aligned} \tag{S9}$$

with $s_j = \frac{(-1)^{(j-1)/2}\Lambda^j}{j!}$ for odd j ($s_j = 0$ for even j) and $c_j = \frac{(-1)^{j/2}\Lambda^j}{j!}$ for even j ($c_j = 0$ for odd j). The summation only contains the terms of j amplitudes ($A_k, A_\ell, \ldots$) whose indices $k, \ell, \ldots$ add up to n. To clarify, for $n = 3$, we sum over $A_1A_1A_1$, A_1A_2, A_2A_1, A_3A_0, A_0A_3 as well as many terms with more A_0's. For every n, we get an infinite number of contributions to each term. The denominator contains at most two terms that depend on n, but the rest is constant and the whole denominator converges for each n; thus we can truncate the denominator, as we have done in Eq. (S9). In contrast, to evaluate the numerator we can take sequentially higher terms in the Taylor expansion (i.e. increase j), find all combinations of j integers that sum to n, and add them to the term of frequency $n\omega_0$.

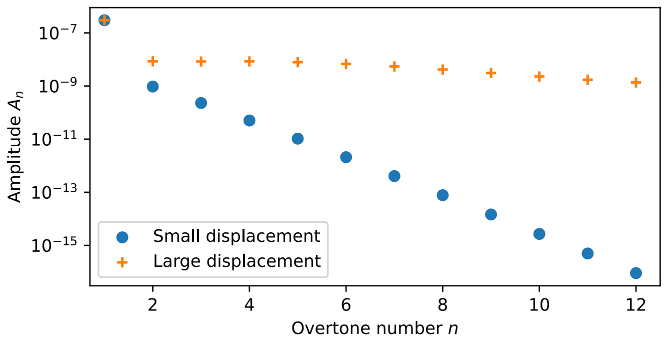

FIG. S2. **Overtone amplitudes.** Numerically evaluated overtone amplitudes A_n in the small- and large-displacement case. The terms $A_0 = 1 \cdot 10^{-7}$ and $A_1 = 3 \cdot 10^{-7}$ are taken constant, with F_o corresponding to 100 pN. For large displacement, the overtones have much higher amplitude than in the small-displacement case. The amplitude also decays more slowly with mode number.

It is difficult to analytically express all the combinations of j integers that sum up to n, so we numerically evaluate the overtone amplitudes in Fig. S2. Here, we take the expansion up to 13th order, which results in close to $2.5 \cdot 10^6$ terms contributing to $n = 12$. We fix the terms with $n = 0, 1$ to a constant value, in the experiment we do not directly apply a resonant drive (A_1) and there are radiation pressure effects that could result in a static position offset (A_0). From the numerical evaluations, we consistently see that for small displacement, the power in each subsequent overtone drops exponentially. However, for large displacement this is no longer true, and the higher-order terms from the expansion cause strong overtones that decrease much slower in amplitude with overtone number. This is reproduced in the experiments shown in the main text.

3. *Numerical simulation of overtones*

So far, we have derived analytically that the addition of an optical $\sin(x)$ non-linearity to a mechanical harmonic oscillator creates components of mechanical motion at integer multiples of the original frequency ω_0. If the displacement is large enough, these overtones have significant amplitude and form a frequency comb. We have made some simplifying assumptions (e.g. $x_{\mathrm{off}} = 0$), which do not necessarily hold in practice. The remedy that, we perform numerical simulations. These allow us to better understand the roles of resonator displacement x, the the optical (dielectrophoretic) force F_{o}, and the position offset x_{off} of the optical potential with respect to the mechanical potential.

First, we investigate the dependence of the frequency comb on displacement x. In Fig. S3**a**, we simulate the motion of the resonator starting from the value initial amplitude x_0 ($v_0 = 0$) indicated in the legend. We include dissipation but it is sufficiently small that the amplitude does not significantly decrease within the simulation time. For small amplitude of motion ($x_0 < x_{\mathrm{off}}$), we cross no optical extrema and only the fundamental mode is visible, no higher overtones appear. Once the motion is large enough to cross one optical extremum (100 nm), several overtones appear with exponentially decreasing power, as derived previously. If the motion is large enough to cross multiple optical extrema ($x_0 > \lambda/4 \simeq 150$ nm), more overtones appear. The lower overtones have approximately equal amplitude (empirically until $\omega = 3x_0/(\lambda/4) \times \omega_0$), since these also interact with the optical potential and get modulated to drive higher overtones. Note that the lower overtones have the same amplitude regardless of the resonator displacement. The higher overtones, above $\omega = 3x_0/(\lambda/4) \times \omega_0$, have an amplitude that decays exponentially.

Secondly, we study the dependence of the frequency comb on dielectrophoretic force F_{o}. For identical initial position x_0, we plot the frequency comb for various values of F_{o} in Fig. S3**b**. For $F_{\mathrm{o}} = 0$ N, we see the dominant fundamental mode but also some higher harmonics (3,5,7,...). These are from the numerical accuracy of our simulation, they do not follow the exponentially decaying trend of the other curves. For increasing values of F_{o}, the amplitude of each of the individual overtones increases linearly, but the exponential fall-off for higher overtone numbers is unchanged. At some point, the non-linearities start to shift the frequency of the modes, but this trap strength is well beyond the regime of our setup ($F_{\mathrm{o}} = 100$ nN corresponds to approximately 6 W of incident laser power).

Finally, we report on the dependence of the frequency comb on position offset x_{off}. In the analytical case, we simplified using $x_{\mathrm{off}} = 0$ nm, retaining only the cosine terms so the even overtones are strong (dark blue). By shifting the optical potential with respect to the mechanical zero by $\lambda/8 = 79$ nm, we see mainly the odd-numbered overtones appear. For this value of x_{off}, we can rewrite the cosine into a sine, such that the Taylor-series expansion only contains odd terms. At other values of x_{off}, we have a weighted average of the two series expansions. In the middle, at $x_{\mathrm{off}} = 35$ nm, we see odd and even numbered modes with approximately equal power.

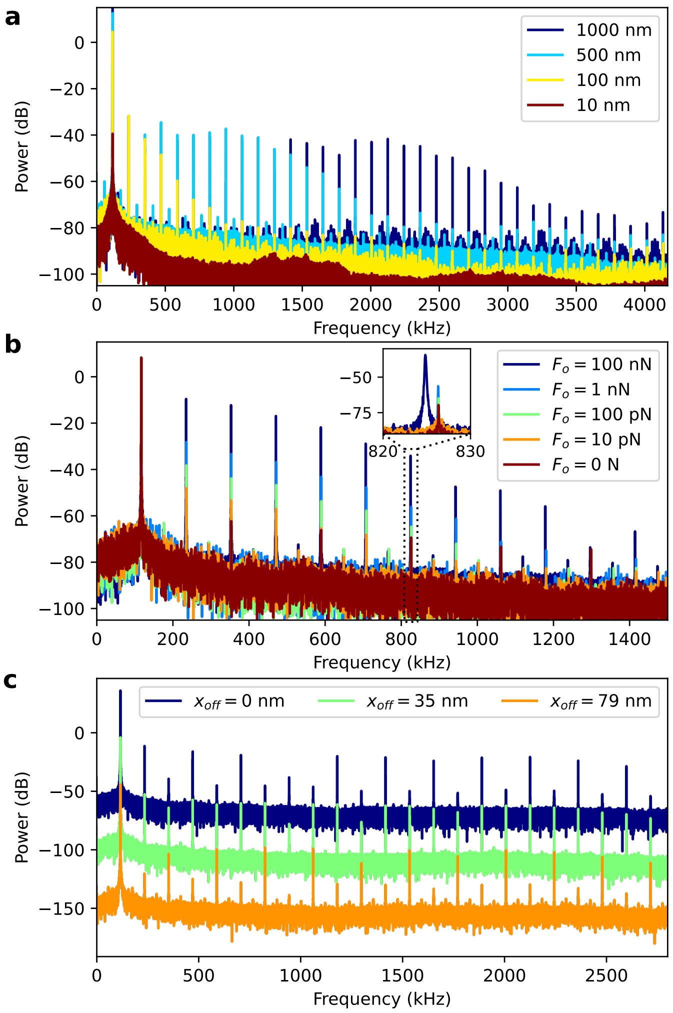

FIG. S3. **a**: Numerical simulation of overtones generated depending on initial amplitude. Parameters $F_{\mathrm{o}} = 100$ pN, $\omega_0 = 2\pi \times 118.049$ kHz, $\gamma = 2\pi \times 0.02$ Hz, $F_{\mathrm{d}} = 0$ N, $x_{\mathrm{off}} = 30$ nm. **b**: Simulation of overtones generated depending on optical force F_{o}, for $x_o = 200$ nm. Other parameters identical to **a**. Inset shows the frequency shift for stronger optical fields. **c**: Simulation of overtones depending on offset x_{off}. For $x_{\mathrm{off}} = 0$ nm, we predominantly drive the even overtones through the cosine-expansion, while for $x_{\mathrm{off}} = \lambda/8 = 79$ nm we predominantly drive the odd overtones through the sine-expansion. At $x_{\mathrm{off}} = 35$ nm, odd and even modes are approximately equal in power. Individual traces are offset vertically, $x_0 = 1000$ nm and other parameters are identical to **a**.

To summarize: The addition of an optical $\sin(x)$ non-linearity to a mechanical harmonic oscillator creates components of mechanical motion at integer multiples of the original frequency ω_0, which we call the overtones of ω_0. These overtones can have significant amplitude, if the mechanical displacement is large enough. The displacement x controls the number of overtones visible, while the dielectrophoretic force F_{o} determines their power relative to the fundamental mode of the comb. Based on the offset between the optical and mechanical potential x_{off}, we can control the relative power in

the even- or odd-numbered overtones. We have thus derived an analytical model for the overtone frequency comb, and expanded our qualitative understanding with numerical simulations.

4. *Exclusion of mechanical nonlinearity*

We have introduced an optical nonlinearity to explain the mechanical frequency combs, but we have not excluded conventional mechanical nonlinearities (e.g. a Duffing term, $\propto x^3$). These nonlinearities can also lead to mechanical frequency combs, even in the absence of other modes to couple to [2, 4]. In Fig. S4**a**, we have simulated and plotted the spectrum of a mechanical resonator with a Duffing term $c_{\text{duff}} x^3$, such that the equation of motion is

$$\ddot{x} + \gamma\dot{x} + \omega_0^2 x + c_{\text{duff}} x^3 = 0. \tag{S10}$$

We start from an initial condition of $x_0 = 500$ nm for all traces. For a sufficiently strong nonlinearity, we see a frequency comb. However, due to the nonlinearity, the frequency is shifted away from ω_0. This is expected behavior for nonlinear resonators, oscillating at ω_0 for small amplitude frequency shifting for larger amplitudes where the nonlinearity becomes dominant. The sign of c_{duff} controls the direction of the frequency shift, and a hardening nonlinearity (increasing frequency) is expected for most mechanical resonators.

In Fig. S4**b**, we have plotted the fundamental mode during the measurement shown also in Fig. 3**e**. At the start of the measurement, the amplitude is small and there is no frequency comb, whereas at the end the amplitude is large and the comb is clear. There is no shift of the frequency of the fundamental mode visible in this measurement, which excludes the explanation of a Duffing nonlinear term being the origin of the comb.

It was proposed by others that strong, higher order nonlinearities (e.g. $\propto x^3$, x^5, x^7, etc.) could be the origin of this comb [2]. However, to reproduce the frequency comb without a similar frequency shift as in the Duffing case, one would need strong nonlinear terms of very high orders. Rather than assuming as many nonlinear terms as we have overtones (up to 35), we have introduced an optical nonlinearity that can explain all overtones from a single effect.

5. *Comparison to other combs*

There have been demonstrations of mechanical frequency combs generated via different mechanisms in literature. For applications, the comb properties are more important than the generation mechanism, and we have summarized a large part of the combs available from literature in Table S1. The entries are sorted by material/geometry platform, and multiple publications with subtle difference in the comb generation mechanism may be present within the individual categories.

The overtone comb of this work has a bandwidth on par with the largest available in literature (limited by the setup), but also has a relatively large resolution. The bandwidth is given by a combination of the maximum displacement and mechanical frequency, both can easily be engineered. We estimate the optothermal parametric drive is limited to $\lesssim 500$ kHz in our membrane, which would constrain the mechanical frequencies that can be operated without an external drive. The frequency resolution could be enhanced by lowering the mechanical frequency (i.e. make the membrane tethers longer and thinner), and can likely be tuned thermally. A further benefit is that the overtone combs are spectrally flat (same amplitude in different overtones), in contrast to other mechanical combs.

The main benefit of the overtone comb is that it does not require an external drive, which is unique for a mechanical comb. This greatly simplifies the necessary setup to operate the comb. In terms of uniformity of the frequency spacing, overtone combs come close to the level of electromechanical combs, where the uniformity is given by the electric drives directly. However, our measured uniformity is limited by the detection setup. Overtone combs are also among the most stable, especially comparing our 6-hour frequency stability (Sec. I H) to the 10 s Allan deviation of Ref. [5].

B. Higher harmonics in LDVs

There are several mechanisms by which spurious higher harmonics can appear in measurements from a LDV [16]. In the following paragraphs, we will discuss these mechanisms and show that we can exclude them as the source of the observed frequency comb. We will treat all parts of the setup shown in Fig. 5: the optical parts, the photodetector, the decoder and the data acquisition.

Multi-path interferences may happen when the LDV laser beam is reflected by more than one surface [17]. The geometry of our system facilitates having multiple reflection sources, since both the trampoline membrane and the substrate contribute to the reflection. We assume an ideal LDV, with an incident electrical field with amplitude E_0, a moving membrane, and a stationary substrate, as shown in Fig. S5**a**. The directly reflected (desired) beam from the membrane has power $R_{\text{SiN}}|E_0|^2$ ($R_{\text{SiN}} = 0.3$). Assuming no scattering ($T + R = 1$, for transmission T and reflection R), the substrate contributes $T_{\text{SiN}}^2 R_{\text{Si}}|E_0|^2 \simeq 0.17|E_0|^2$ ($R_{\text{Si}} = 0.35$). Only the membrane contribution has a Doppler shift due to its motion, $+v$. Neglecting effects from beam divergence, multiply-reflected beams contribute $T_{\text{SiN}}^2 R_{\text{Si}}^2 R_{\text{SiN}}$, $T_{\text{SiN}}^2 R_{\text{Si}}^3 R_{\text{SiN}}^2$, and so on. These terms have Doppler shifts with the opposite sign to the directly reflected beam, as they interact with the membrane from the opposite direction. The first term has a Doppler shift equal in magnitude to the desired signal ($-v$), while subsequent terms have multiple times the Doppler shift ($-2v, -3v, ...$). These terms thus directly affect the magnitude of the velocity that the LDV senses. For harmonic membrane motion $v(t) = v_0 \cos(\omega_0 t)$, the multiply-reflected beams lead to an error in v_0, but their Doppler shift does not contribute directly to higher harmonics of the observed membrane motion.

In a non-ideal laser Doppler vibrometer, multi-path inter-

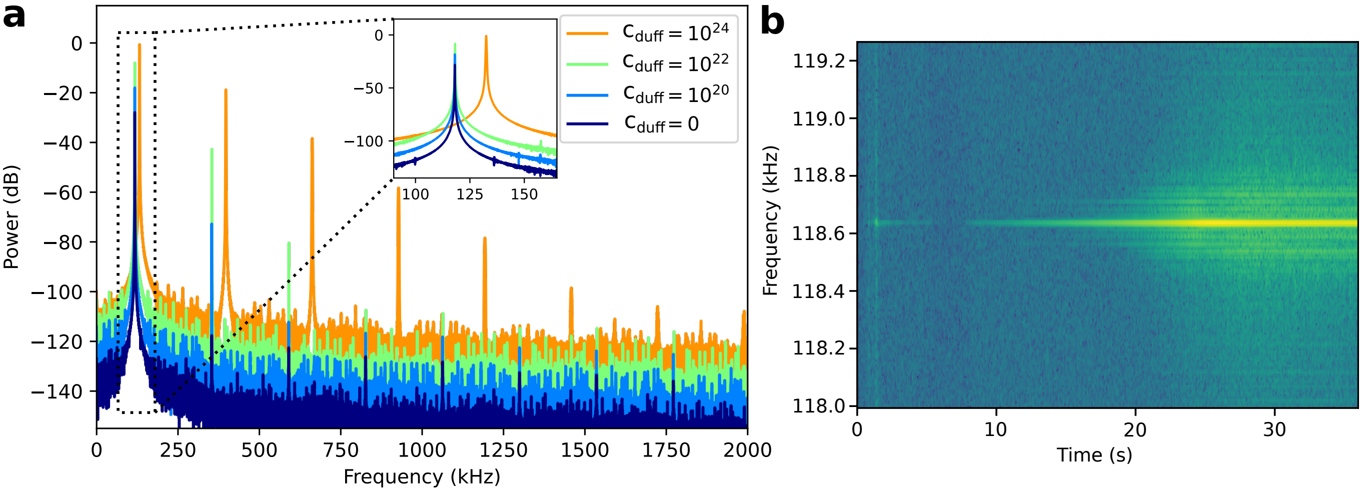

FIG. S4. **Frequency comb by mechanical nonlinearity.** **a**: Simulated displacement spectrum of a mechanical resonator with a Duffing nonlinearity. All traces share the same initial displacement, x_0 = 500 nm, with different Duffing coefficients. For a sufficiently large nonlinearity, the frequency comb appears, but the frequency of the fundamental mode (and comb spacing) is changed. **b**: Measured frequency of the fundamental mode of Fig. 3**e**, showing no shift in frequency between low displacement (no comb) and high displacement (comb).

Type	Bandwidth (Hz)	Resolution (Hz)	Ext. drive power (dBm)	Uniformity (-)	Stability (-)	Footprint (μm^2)
Electromechanical [6]	20 – 100	0.005 – 10	−15 [7]	$1 \cdot 10^{-9}$		$3 \cdot 1$ – $150 \cdot 50$
Nanostrings [8]	10 – 25 ($\times 10^3$)	0.5 – 10 ($\times 10^3$)	−2.5 [4]			$55 \cdot 0.27$
Free-beam [9]	60 – 150 ($\times 10^3$)	2 – 10 ($\times 10^3$) [10]	5	$5 \cdot 10^{-6}$ [5]	$1 \cdot 10^{-8}$ [5]	$1100 \cdot 350$
Coupled beams [11]	10 – 200	1 – 30 [12]	−26			$500 \cdot 50$
Bulk acoustic [13]	20	0.7 – 2	−67			$23000 \cdot 1000$
Circular 2D [14, 15]	2 – 11 ($\times 10^6$)	75 – 400 ($\times 10^3$)	10			5 – 8 (diameter)
This work	4.2 ($\times 10^6$)	118 – 359 ($\times 10^3$)	None	$4.7 \cdot 10^{-8}$	$7.5 \cdot 10^{-10}$	$750 \cdot 750$

TABLE S1. **Mechanical frequency comb performance.** Overview of reported mechanical frequency comb properties from literature, organized by platform (irrespective of comb mechanism). The overtone comb has a bandwidth (frequency span) on par with the largest combs, but also a relatively large resolution (frequency spacing). We report the absolute frequency stability over a 6-hour period (see SI Sec. I H), whereas [5] computes the Allan deviation over a 10 s period. Our work is the only mechanical frequency comb that does not require any external drive.

ferences can be identified by presence of ripples and spikes in the velocity signal. We can compare the measured velocity signals with simulated signals using the model of [17]. For a harmonic oscillator with ω_0 = 118 kHz, maximum displacement amplitude $x_{\max}$, the demodulated velocity signal has the form

$$v(t) = -\frac{1}{2}\omega_0 x_{\max}\left(\frac{\theta_2 - 1}{2\theta\cos\left(\frac{4\pi}{\lambda}\cos(\omega t) + \Delta\phi\right) + \theta^2 + 1}\right)\sin(\omega t). \tag{S11}$$

Here, θ is the electric field ratio of the correct and the unwanted reflected signals ($\theta = \infty$ for an ideal vibrometer), $\Delta\phi$ is the phase offset between the two beams (constant). In the worst-case scenario ($R_{\mathrm{SiN}} = 0.3$, $R_{\mathrm{Si}} = 0.35$, no scattering losses), $\theta \simeq 1.33$. The unwanted beam likely suffers from scattering losses more than the correct beam, since its path crosses the Si_3N_4 domain twice and reflects of the etch-roughened Si surface (see Fig. 5**d**).

We simulate the velocity signal with multi-path interferences, and compare it with the observed velocity signal in Fig. S6. The simulated signal of Fig. S6**b** ($\theta = 10$) has the characteristic ripples of multi-path interference. By comparing the simulated signal to the measured signal (Fig. S6**a**), one sees that the characteristic ripples are absent. While it is unlikely that the multi-path interference effect is completely absent in our setup, it can be corrected by a combination of amplitude- and phase-locked loops [18]. Either way, the observed velocity signal does not correspond to multi-path interference effects.

We can further exclude multi-path interferences as the source of the comb by measuring outside the membrane, on the Si chip. At the position indicated on the microscope image of Fig. S6**d**, the Si and Si_3N_4 layers are touching and thus have the same motion. The multi-path interference as described in Ref. [17] does not happen. We use a second beam ('drive beam' in Fig. S6**d**) to generate a comb of the fundamental mode of the membrane. At the measurement position outside the membrane, we can detect this comb, red curve in Fig. S6**c**. For verification, without the drive beam present (blue curve), no comb is detected while the fundamental mode is visible. Thus we can exclude multi-path interference effects as the source of the observed frequency combs.

High-aperture effects can occur when the LDV is operated with a microscope objective, based on the Guoy phase delay of the optical beam [16, 19]. The majority of the measure-

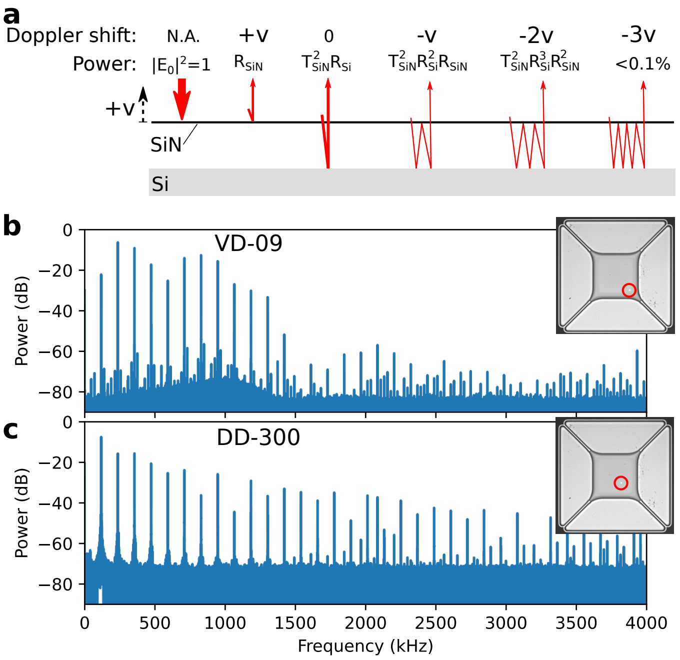

FIG. S5. **Sources of higher harmonics.** **a**: Schematic of reflected components of a single optical beam on our device (red arrows). For each component, the Doppler shift due to membrane velocity v is shown, as well as the relative power. **b**: Frequency comb measured using the VD-09 decoder (maximum bandwidth set to 1.5 MHz to allow maximum detectable amplitude). Inset shows microscope image with laser position highlighted by the red circle. **c**: Frequency comb measured using the DD-300 decoder, measured on the same device at a slightly different position.

ments in this work were done using a Mitutoyo Plan APO 5x objective with a numerical aperture of 0.14. With this numerical aperture, the amplitude error and harmonic distortion should be limited to $< 1\%$ [16]. Additionally, we compare the frequency combs obtained from the same device using different lenses (different numerical apertures) in Sec. I G, which show no relation between the numerical aperture and frequency comb. Thus we can exclude high-aperture effects as the source of the observed frequency comb.

The electronic parts of the LDV (sketched in Fig. 5**a**) may contribute to a nonlinear response that would generate harmonics [16]. We record frequencies well below the 20 MHz specified maximum frequency of the detector, thus this component should not contribute higher harmonics.

In a conventional heterodyne interferometer, harmonics of a signal would naturally arise for sufficiently large displacement. The interferometer phase is linear only in a limited regime, typically $x \ll \lambda$. However, a Doppler vibrometer measures the frequency shift of the reflected light rather than its phase. The frequency shift can be extracted with a frequency-to-voltage converter, in the LDV decoder. This allows an LDV to measure velocities associated with displacements much larger than the optical wavelength, without suffering from the harmonics expected in a displacement interferometer.

There are two LDV decoders available in our setup, the VD-09 velocity decoder and the DD-300 displacement decoder. The VD-09 has a lower maximum operating frequency (1.5 − 2.5 MHz), while the DD-300 has a lower detection maximum. In Fig. S5**b,c**, we show the frequency combs measured from the same device with the different decoders. These measurements show that the frequency comb is not a decoder artifact. Thus we can exclude the electronic parts of the measurement setup as a source of the observed frequency comb.

In summary, we have described common mechanisms that yield higher harmonics in laser Doppler vibrometer measurements. All these mechanisms can be excluded as the cause of the frequency combs, by qualitative arguments and the measurements shown in Figs. S5, S6, and S17. In combination with the fact that there is a visible change in the membrane when the frequency comb occurs (Fig. S15), we conclude that the observed mechanical frequency comb is a real, physical phenomenon and not an artifact of the measurement setup.

C. Membrane in an optical trap

Optical traps can be used to confine small particles, as the light exerts a force proportional to the gradient of the optical intensity [20, 21]. For uncharged dielectric particles, the electrical field polarizes the particles, and any change in field (from a gradient) will result in a dielectrophoretic force acting on that particle [22]. In this section, we will simulate a laser incident on a dielectric (Si_3N_4) membrane, and evaluate the dielectrophoretic and radiation pressure forces. We will show that the optical gradient is sufficient for the dielectrophoretic force to affect the mechanical motion, while the radiation pressure force does not affect the dynamics significantly.

1. Dielectrophoretic force

We simulate the physical system consisting of a $t \simeq 80$ nm thin Si_3N_4 slab suspended in air above a much thicker Si slab, shown in Fig. S7**a**. We study a 2D-axisymmetric domain centered around our laser spot, which is incident from the top of the figure. We assume that our laser Doppler vibrometer emits a Gaussian beam for convenience. For a regular optical trap, the optical gradient and thus the force exerted is weakest along the laser propagation direction, while it is stronger in the plane normal to the propagation direction. However, the Si backplane in our geometry reflects part of the light and produces a standing wave (Fig. S7**a**), producing a periodic optical intensity. Combined with the fact that the membranes are much larger than the optical gradient laterally ($\sim 750 \times 750$ μm^2), we have a one-dimensional counterpropagating-wave optical trap in the out-of-plane direction. A simple (unpatterned) Si backplane has been shown before to be sufficient to create an interference pattern for such an optical trap [23].

We consider only motion in the out-of-plane direction of the membrane, so we are interested in the gradient of the electric field norm in the y-direction (Fig. S7**a**). The total force

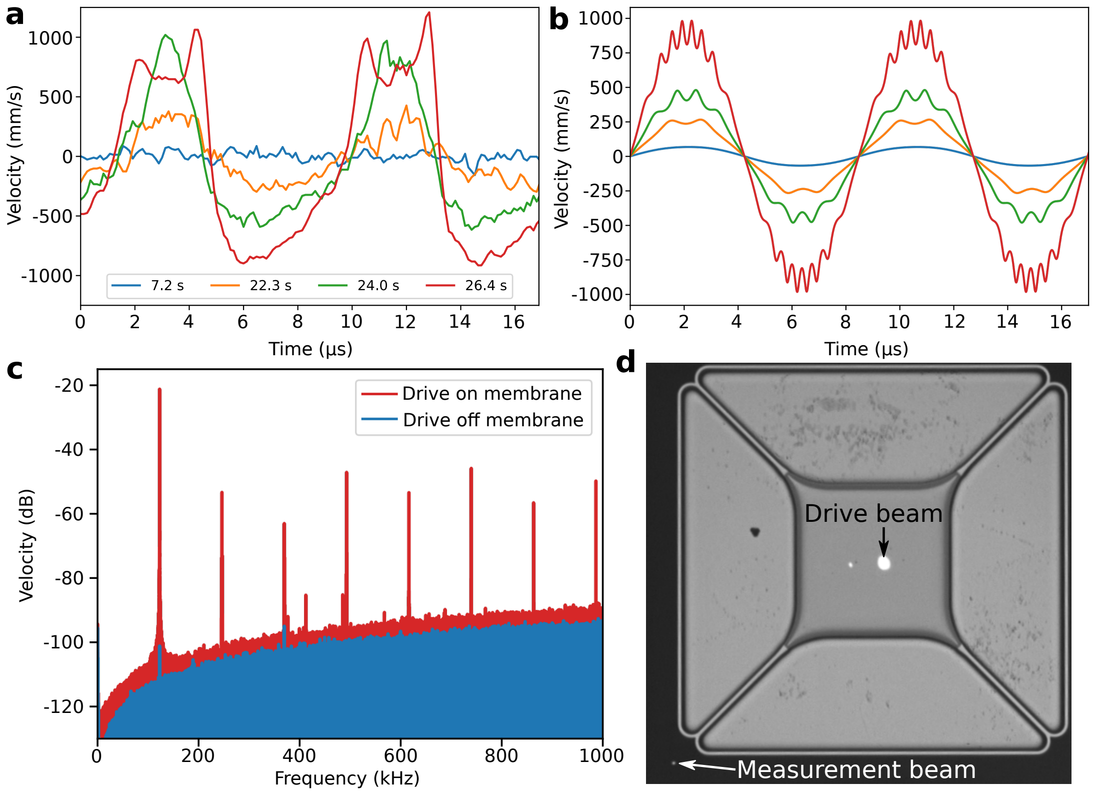

FIG. S6. **Exluding multi-path interferences.** **a**: Measured velocity signal for different times in Fig. 4**b**. The data has been smoothed with a first order Savitzky-Golay filter of width 3, to emphasize the presence of ripples. **b**: Simulated velocity signal of a resonator with frequency 118 kHz with multi-path interferences present. The characteristic ripples of the interference are visible at the extrema of the velocity curve in **b**, but absent in **a**. **c**: Measured velocity spectrum outside the membrane, on chip. A second (drive) beam generates a frequency comb of the membrane's fundamental mode, which we detect at the position indicated on the microscope image **d**. Without this beam on the membrane, no comb is observed.

F_{de} induced by the optics on the dielectric Si_3N_4 structure can then be found by integrating this gradient over the membrane domain,

$$F_{de} = \int_{V_{SiN}} \alpha \frac{\partial |E|^2}{\partial y} dV, \quad \text{(S12)}$$

with α the polarizability. For a linear isotropic material, the constant $\alpha = P_x/E_x$ can be found from the simulation by dividing the polarization in any direction P_x by the electric field in that direction E_x.

We need relate the equation for F_{de} (units of force) to the coefficient F_o (units of force) used in Eq. 1. The expression for F_{de} depends on the position of the resonator with respect to the Si backplane, Fig. S7**b**, whereas F_o is a constant. $F_{de}(x)$ in Fig. S7**b** shows the periodic behavior expected from an optical trap (i.e. periodic sign change), as well as a slight asymmetry due to radiation pressure. It shares the periodicity that we have separately introduced in Eq. 1 in the main text, $\sin\left(\frac{4\pi}{\lambda}(x - x_{off})\right)$, but does not exactly follow the same curve due to the finite size (thickness) of the membrane with respect to the wavelength. To simplify the dynamical calculations, we have thus chosen to separate out the periodic behavior and leave it proportional to the optical intensity, $\sin\left(\frac{4\pi}{\lambda}(x - x_{off})\right)$, and keep F_o as a constant equal to the maximum of $|F_{de}(x)|$. Thus for a $t = 80$ nm membrane subject to our 3.6 mW laser, we expect a maximum out-of-plane force in the order of 60 pN. This confirms the values of F_o used in the numerical simulations in the main text and in Sec. I A, which show that the dynamics of our membrane can be significantly altered by the presence of the optical field.

The different curves of Fig. S7**b** illustrate that the total optical force exerted on the membrane does not decrease monotonically with membrane thickness. When we calculate the effective strength of the trap (peak-to-peak force difference divided by the thickness of the membrane, in Fig. S7**c**), we see that it decreases for increasing Si_3N_4 thickness. There are minima around $t = \lambda/(2n_{SiN})$ where n_{SiN} the index of refraction; the half-integer number of optical periods leads to a total cancellation of the net force on the membrane F_{de}.

2. *Radiation pressure force*

In an optical cavity, the interaction between radiation pressure and a mechanically compliant element can lead to optomechanical frequency combs [24]. The radiation pressure of an optical cavity coupled to a mechanical resonator can result in an effective cubic non-linearity, and by sufficiently

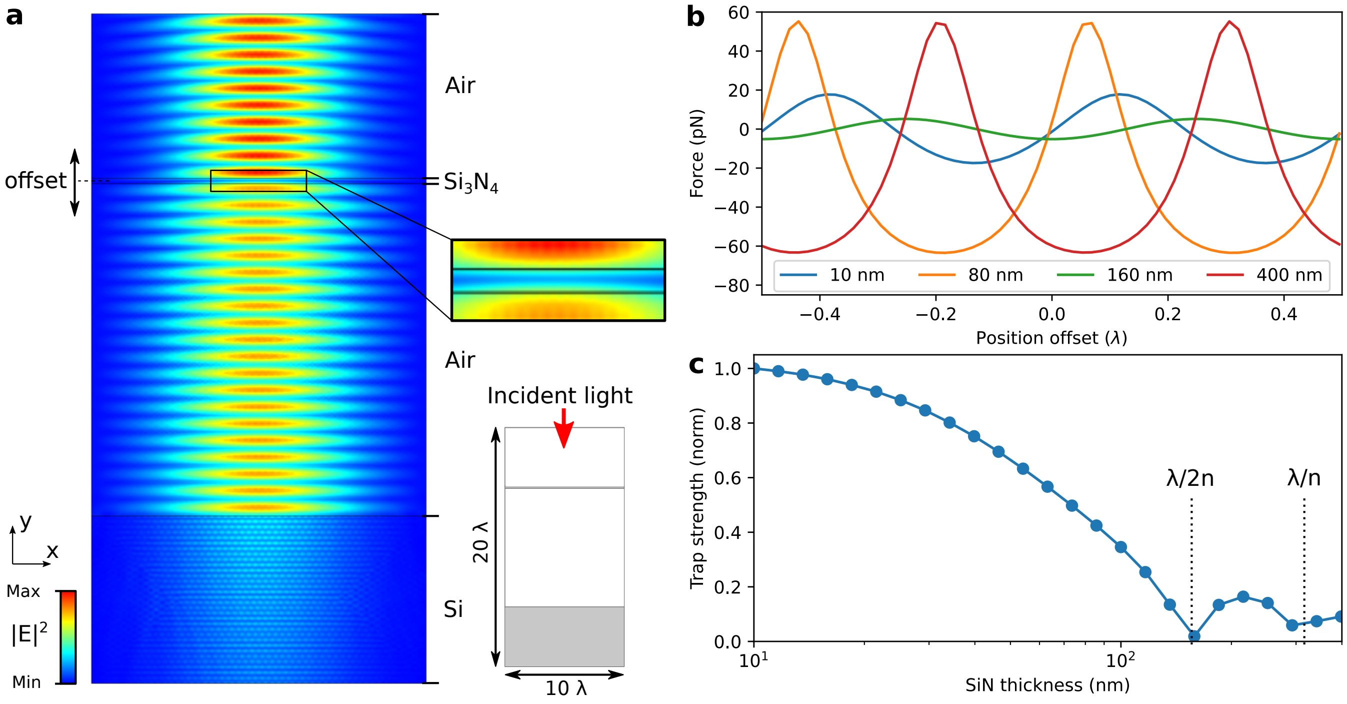

FIG. S7. **Simulation of optical trap. a:** Electric field norm of incident 633 nm light on 100 nm Si_3N_4 membrane suspended ~6 µm above a Si backplane. The reflected light forms a standing wave pattern. Zoom shows center of the Si_3N_4 membrane. Bottom inset shows the simulation geometry schematically. **b:** Force exerted by the optical field on the Si_3N_4 domain as the position of the membrane is varied. The colors represent different membrane thicknesses. **c:** Normalized strength of the trap for different Si_3N_4 thicknesses.

driving the optical cavity one can bring the system in the self-oscillation regime. This creates an optical frequency comb with a spacing equal to the mechanical frequency. We must thus distinguish the frequency combs from the dielectrophoretic force from those originating from the radiation pressure.

We follow the analysis of Miri et al. [24] as they analyze the stability of a mechanically compliant end-mirror optomechanically coupled to an optical cavity. Our Si_3N_4 membrane functions as the compliant end mirror, while the Si substrate is the other (fixed) end mirror. For a given set of parameters, we evaluate whether the frequency comb will appear through equations (9a,b) of [24], which amounts to checking the Routh-Hurwitz stability criterion. For completion, the conditions for stability are

$$\begin{aligned} 3\mathcal{G}^2 + 4\Delta\mathcal{G} + \Delta^2 + \kappa^2/4 > 0, \\ \mathcal{G}^4 + d_1\mathcal{G}^3 + d_2\mathcal{G}^2 + d_3\mathcal{G} + d_4 > 0, \end{aligned} \tag{S13}$$

with coefficients

$$\begin{aligned} d_1 =& 4\Delta, \\ d_2 =& \gamma\kappa + 6\Delta^2 + \gamma^2 - 6\omega_0^2 + \frac{\kappa^2}{2} - \frac{2\gamma\omega_0^2}{\kappa} - \frac{2\omega_0^2\kappa}{\gamma}, \\ d_3 =& \Delta\left(2\gamma\kappa + 4\Delta^2 + 2\gamma^2 - 8\omega_0^2 + \kappa^2 - \frac{2\gamma\omega_0^2}{\kappa} - \frac{2\omega_0^2\kappa}{\gamma}\right), \\ d_4 =& \Delta^2\left(\Delta^2 + \gamma^2 + \gamma\kappa - 2\omega_0^2 + \frac{\kappa^2}{2}\right) + \frac{\gamma^2\kappa^2}{4} \\ & + \gamma\omega_0^2\kappa + \frac{\gamma\kappa^3}{4} + \left(\omega_0^2 + \frac{\kappa^2}{4}\right)^2. \end{aligned} \tag{S14}$$

The key quantity is the static optomechanical frequency shift $\mathcal{G} = \frac{2g_0^2}{\omega_0}|a|^2$, with $g_0 = 2\pi \times 1$ Hz the vacuum optomechanical coupling strength typical for these membranes [25]. Additionally, $a = \left(\frac{\kappa P_\ell/4\hbar\omega_\ell}{(\kappa/2+i\Delta)}\right)^{1/2}$ is the average optical field amplitude in the cavity, determined by the cavity decay rate κ, laser power and frequency P_ℓ, ω_ℓ and detuning Δ. Finally, the mechanical frequency $\omega_0 = 2\pi \times 120$ kHz and linewidth $\gamma = 2\pi \times 0.1$ Hz are rounded from the values measured and reported in the main text.

To estimate κ, we calculate the finesse of the Fabry-Pérot cavity formed by a 10 µm distance between an $R = 0.3$ Si_3N_4 and an $R = 0.35$ Si backplane. This is a low estimate for κ, as the Si surface is rough from fabrication (Fig. 5**d**), and we do not consider additional losses from that. We obtain $\kappa \sim 2\pi \times 430$ GHz. We assume the optimal detuning to reach

the frequency comb regime, which is around $\Delta = -\kappa/4$. Combining these values, we can evaluate the expressions for the stability and determine a minimum input laser power to reach the frequency comb regime. This is at approximately 1.5 kW, several orders of magnitude larger than the 3.6 mW measured laser power output of our laser Doppler vibrometer. Thus the optomechanical (radiation pressure) instability is not the origin of our frequency combs.

In conclusion, we have simulated the behavior of a Si_3N_4 membrane subject to an incident laser. Due to the Si backplane, reflected light forms a standing wave which leads to a counterpropagating optical trap. We have calculated the force exerted on the membrane by the optical trap, its dielectrophoretic part is sufficient to generate the overtone frequency comb as described in the main text. We have also verified that the optical cavity formed between the Si_3N_4 membrane and Si backplane is not good enough to lead to an optomechanical frequency comb based on the radiation pressure.

D. Optothermal parametric driving

Driving the trampoline resonators to sufficiently large amplitudes to see the frequency comb from the optical trapping potential requires a strong driving mechanism. We have previously used a piezo shaker to drive these membranes [26] (also described in Sec. I E), but for the majority of this work we use a optothermal parametric drive mechanism where the membrane reaches the self-oscillation regime [27]. That means that we can use a continuous-wave laser without power or frequency modulation, and do not require any additional signal or connection to the chip containing the resonator.

In the following subsections, we study the optothermal parametric driving. Since it is based on absorption, which is normally not too large for $\lambda = 633$ nm in Si_3N_4, we first show the absorption is enhanced by our photonic crystal structure. Then, we simulate the slow (> 0.1 s) thermal dynamics from steady state absorption and the fast ($\ll 0.1$ s) thermal dynamics due to modulated intensity from the standing wave optical field. Finally, we compare the simulated changes in temperature to the optothermal self-oscillation theory [27] and other effects from literature.

1. *Absorption in the photonic crystal*

Si_3N_4 is a often-used optical material because of its low absorption at telecom wavelengths, with typical absorption coefficients $\mu = 1-2$ cm^{-1} at 1550 nm [28]. However at 633 nm, absorption is higher and sensitive to deposition parameters (reports vary from $\mu = 2$ cm^{-1} [29] to $\mu = 5000$ cm^{-1} [30]. Works using a similar setup but low-stress Si_3N_4 measured 0.5 % absorption for a 50 nm film ($\mu = 100$ cm^{-1}) [31], so for simulation purposes we assume 1 % for our 80 nm film. It is difficult to verify the accuracy of this assumption, especially since our trampoline resonators are patterned with an array of circular holes that form a photonic crystal [32] and previously cited absorption coefficients cover unpatterned films. These works suggest absorption should be dependent mainly on material properties (Si_3N_4 deposition parameters) thus be the same for all membranes on a chip. However, we experience quite a variance in the response of our membranes to our laser beam (SI Sec. I G), and also depending on the exact positioning of the laser on the membrane, so we propose a different effect.

Photonic crystal patterns can be designed to yield a certain reflectivity at a target wavelength [32], but for the resonators in this work the photonic crystal functions as a mechanism to accurately control the resonance frequency [26], as well as allowing their smooth undercut. Due to this, the photonic crystal parameters (lattice spacing $a = 1356$ nm, hole radius $r = 481$ nm) are larger than the wavelength, which allows standing waves in the Si_3N_4 domain that couple to waves incident on the plane of the membrane. These modes enhance the field inside the Si_3N_4 and thus could increase the absorption while being subject to fabrication imperfections such that not all membranes or locations on membranes absorb equally, leading to the variance in the observed optothermal self-oscillation.

We simulate the behavior of the photonic crystal in COMSOL by taking a unit cell with the photonic crystal parameters (a, r, and Si_3N_4 thickness $t = 100$ nm), as shown in Fig. S8**a,d**. We use $n = 2.016$ for the Si_3N_4 domain and $n = 1$ for vacuum. Parallel to the plane of the membrane we define two periodic ports, for input (reflection) and output (transmission), which are located sufficiently far away from the Si_3N_4 domain. These ports border a perfectly-matched layer that absorbs scattered light that is not absorbed by the periodic ports. The incident wave is an plane wave with normal incidence to the slab, with a polarization in the $x + y$ direction, and the entire simulation domain has periodic boundary conditions in x and y directions (brown lines in Fig. S8**a**.

From this simulation, we find that a standing wave pattern forms in the plane of the Si_3N_4 membrane, shown in Fig. S8**b**. When we evaluate the reflection and transmission of this photonic crystal, we see only a small feature around $\lambda = 630.8$ nm, Fig. S8**c**. However, the field integrated over the Si_3N_4 domain is much enhanced. These modes are sensitive to material thickness, a and r, and not unique to the parameters chosen for our simulation. We propose that standing waves of this form cause strong(er) absorption of light in some of our structures, depending on fabrication imperfections and precise laser position.

2. *Slow thermal behavior*

To build our understanding of the effects of the absorbed light, we simulate the effect of a heat source on the photonic crystal membrane (Fig. S9**a**). The membrane structure is symmetric, so we can reduce the simulation domain to only a quarter of a membrane. The outer edges of the domain connect the suspended Si_3N_4 connects to the (much more massive) Si substrate, so we apply a fixed temperature condition (purple). The heating due to the laser light is considered as an incident heat flux in the center of the membrane (orange), defined via the

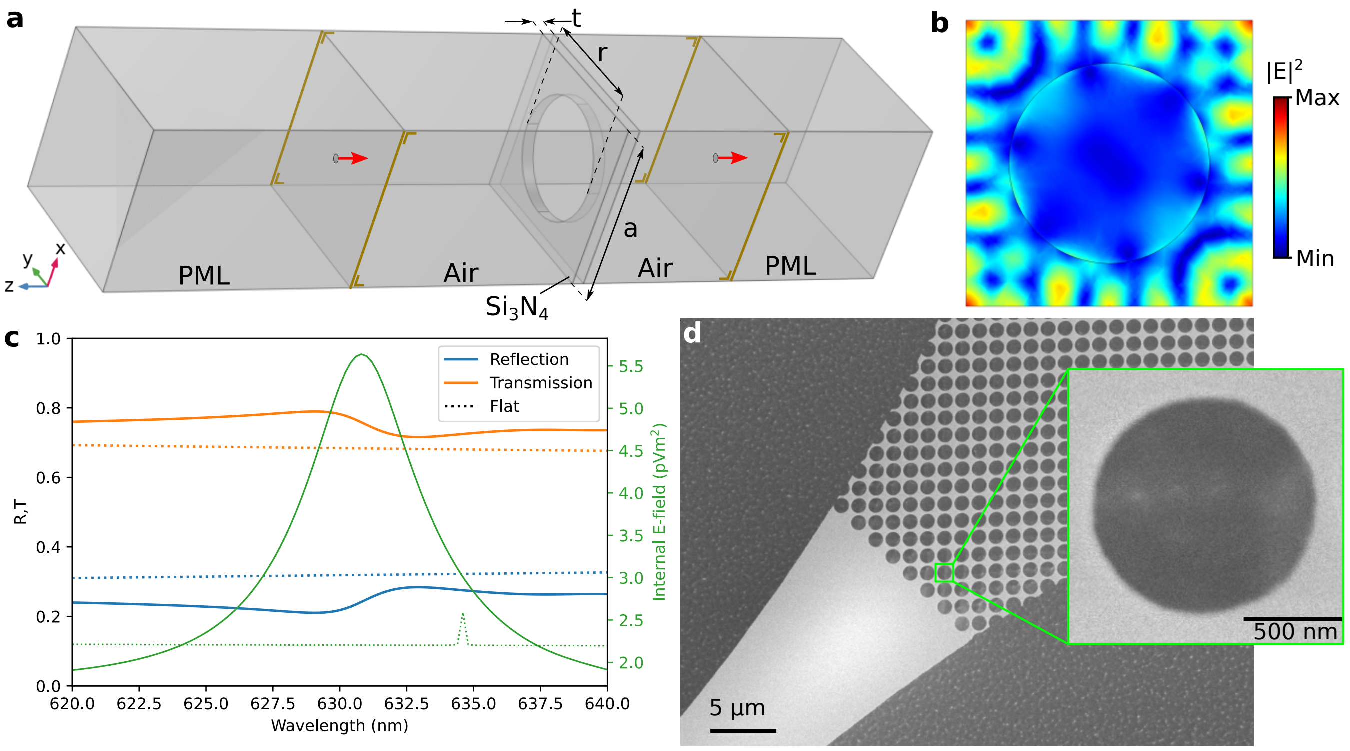

FIG. S8. **Optical behavior of photonic crystal. a:** Schematic of simulation setup, consisting of a Si_3N_4 photonic crystal unit cell in air, with a plane wave incident in the out-of-plane direction (red arrows). There are periodic boundary conditions on the outer surfaces, with the brown markings indicating one of the surface pairs. **b:** Norm of the E-field along the mid-surface of the Si_3N_4 domain, showing a standing wave for $\lambda = 630.8$ nm. **c:** Reflection and transmission of the photonic crystal as a function of wavelength, and that of a flat Si_3N_4 surface for comparison (dotted lines). The feature in the reflection/transmission is correlated with the mode of the photonic crystal shown in **b**, indicated by the total E-field integrated over the Si_3N_4 domain (green). This feature is also absent for an unpatterned surface (green, dotted). **d**: SEM image of photonic crystal at the membrane edge, with inset showing single hole.

total deposited heat as $P\mu_{Si_3N_4}$ with laser power $P = 3.6$ mW and absorption coefficient $\mu_{Si_3N_4} = 0.01$. The heat can be lost through the fixed temperature boundary condition at the outer edges of the domain, but also through radiative transfer in the out-of-plane direction, where we assume the environment is at room temperature. We use a surface emissivity of 0.1, which is valid for room temperature and a film thickness of 100 nm [33], and noticeably different than the 0.6 – 0.9 value used for bulk Si_3N_4.

The choice of material parameters of Si_3N_4 is important for our simulation. The Young's Modulus $E = 250$ GPa, Poisson's ratio $\nu = 0.23$ and density $\rho = 3100$ kg m^{-3} are relatively well-known, and the fabrication pre-stress is known to be 1.0 GPa. The thermal properties reported in literature vary (see [31] and references therein), we take the specific heat $C_p = 700$ J kg^{-1} K^{-1} and thermal expansion coefficient $\Upsilon = 2.3 \times 10^{-6}$ K^{-1}. Literature values for the thermal conductivity vary between $k = 0.34$ W m^{-1} K^{-1} [34] and $k = 20$ W m^{-1} K^{-1} [35]. We simulate using both values and plot the results as solid ($k = 20$ W m^{-1} K^{-1}) or dashed lines ($k = 2$ W m^{-1} K^{-1}) in Fig. S9**b,c,d**.

The simulation results show that for both thermal conductivities, the temperature (Fig. S9**b**) reaches a steady state after $\lesssim 0.3$s, though the temperature is significantly higher for the low thermal conductivity case (470 K) than for the high thermal conductivity case (349 K). Due to this, the material expands by 20 (45) nm for the high (low) thermal conductivity case ((Fig. S9**c**). On a lateral dimension of 150 µm of the membrane this is negligible as it corresponds to approximately a 0.2 (0.4) nm increase in the lattice spacing a. Because of the width of the pad (middle of membrane) with respect to the tether, the stress in the pad is strongly reduced from the initial film stress (1 GPa), to $<$ 20 MPa. By the heating, this can be reduced to close to zero stress (compare yellow-dashed line in Fig. S9**d** to the blue line). Overall, these changes are not so significant and unlikely to change the dynamics of the membrane by themselves.

3. *Fast thermal behavior*

After simulating the steady state in temperature, material deformation and stress described in the previous subsection, we will now include the time-modulated optical intensity. We assume that the resonator moves initially in its fundamental mode at 120 kHz, so the optical field intensity is modulated by the sin term of Eq. 1. We make sure to keep the time-averaged power the same as in our steady-state simulation. We

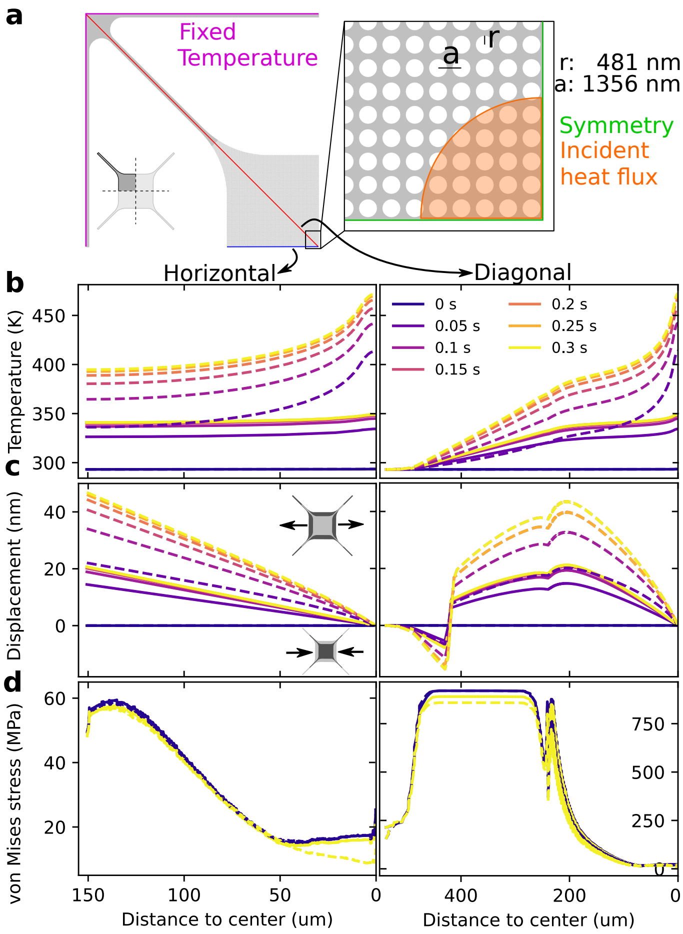

FIG. S9. **Thermal behavior of photonic crystal.** **a:** Schematic of simulation of the laser heating the suspended membrane. Boundary conditions and geometry are described in the text. Results are extracted along two linecuts, horizontal (left column) and diagonal (right column) in **b, c, d**. Starting from room temperature, an incident heat flux representing absorption from the laser light heats up the membrane over time. Colors indicate time, solid lines assume thermal conductivity $k = 20\,\mathrm{W\,m^{-1}\,K^{-1}}$, dashed lines use $k = 2\,\mathrm{W\,m^{-1}\,K^{-1}}$. **b:** Temperature along the linecuts shows heating in the center, which settles within 0.3 s. **c** Displacement of the geometry away from (positve) or towards (negative) the center of the domain due to thermal expansion. The maximum displacement is ~ 45 nm. **d:** Von Mises stress (smoothed, only first and last timestep shown). Left and right panel have different y-axis scale.

simulate a full period, and plot the incident heat flux (grey) and resulting temperature averaged over circular domains at various distances from the center (colors) in Fig. S10**a**.

The incident heat flux modulates the temperature of the membrane with an amplitude of about 0.3 K and a delay on the order of a microsecond. This delay is smallest in the domain where the heat flux is directly incident (blue), and increases as we move further away from the center (orange through red). The change in temperature also decreases as we move further away, which indicates that the effect is local to the laser beam spot. We can also extract the average von Mises stress, Fig. S10**b**, according to

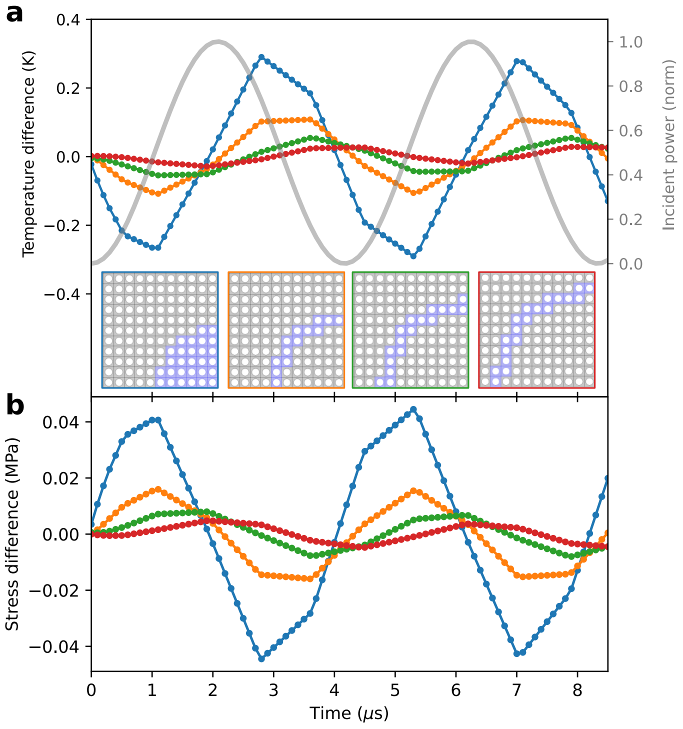

FIG. S10. **Fast thermal dynamics.** **a:** Incident laser power (grey) and average temperature difference with respect to steady state (≃ 348 K for 120 kHz motion through standing wave optical trap field. Colors represent integration over domains depicted in the insets. **b:** Resulting stress change with respect to steady state.

$\sigma_{\mathrm{avg}} = 1/\sqrt{2}\sqrt{(\sigma_{xx} - \sigma_{yy})^2 + 6\sigma_{xy}}$, with $\sigma_{xx,yy,xy}$ representing the stress components in the respective coordinates. This shows a clear inverse correlation with the temperature, and follows the trend of decreasing in amplitude and increasing in delay as we move further away from the incident laser beam spot.

The resulting stress difference is relatively small compared to the total local steady-state stress (~ 24 MPa). However, it can forms a parametric driving mechanism since stress is modulated at double the frequency of the original motion, guaranteed by the optical standing wave. This optothermal parametric driving is thus automatically frequency-matched to the dominant motion. The delay between heat flux and stress suggests that this mechanism of driving is limited in our case to $\lesssim$ 500 kHz, which corroborates with the fact that only observe combs based on the first few eigenmodes.

4. *Self-oscillation*

We have shown so far that the motion of the membrane through the optical standing wave results in a modulated stress via the absorption. However, this alone is not sufficient to drive the resonator to larger amplitudes since dissipation is also present (though small). To estimate whether this effect is strong enough to bring our resonator into limit-cycle oscillations under a $P \lesssim 3$ mW drive, we follow the model of [27].

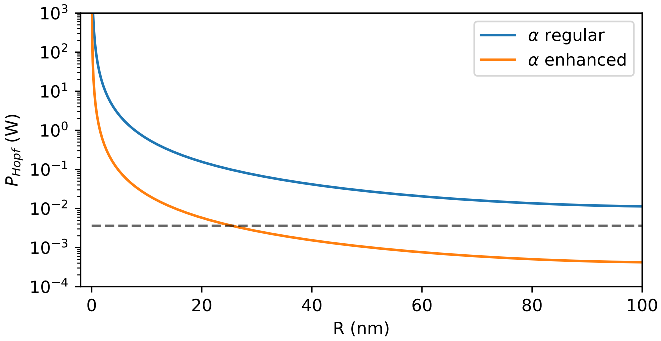

FIG. S11. Simulated optical power necessary to create a limit-cycle of amplitude R, using the absorption expected for a bare Si_3N_4 membrane, and with one enhanced by absorption through internal standing waves due to the photonic crystal. Black dashed lines indicate maximum laser power used in experiments, 3.6 mW.

There, the authors derive the minimum incident laser power to bring a resonator into the self-oscillation regime (Hopf bifurcation),

$$P_{\mathrm{Hopf}} = \frac{3(B^2+1)(B^2+4)}{2\pi^2 AQ\psi K}, \tag{S15}$$

where

$$\begin{aligned} K &= \eta_1\lambda^4R^4 + \eta_2\lambda^2R^2 + \eta_3, \\ \eta_1 &= -4\pi^2(B^2+1)C, \\ \eta_2 &= -24\pi^2(B^2+1)x_{\mathrm{off}}^2C/\lambda^2 - 24\pi^2(B^2+4)x_{\mathrm{off}}D/\lambda \\ &\quad + 3(B^2+1)C, \\ \eta_3 &= -4D(B^2+4)(8\pi^2x_{\mathrm{off}}^2/\lambda^2 - 3)x_{\mathrm{off}}/\lambda. \end{aligned} \tag{S16}$$

Here, A describes the heating due to the laser (units K W^{-1}), B is a dimensionless constant, C represents the stiffness change due to temperature (units K^{-1}), D is the optothermal forcing term (units K^{-1}), Q is the mechanical Q-factor of our resonator, and ψ is the fraction of optical field that forms a standing wave. These equations give the laser power necessary to create a limit-cycle of amplitude R.

The authors of [27] give helpful guide on how to estimate the parameters of Eq. (S15) from the simulations of Secs. I D 3 and I D 2. To estimate the thermal parameters A and B, we require the steady state temperature at the point of illumination (center) of Fig. S9, T_{dc} (difference with respect to the room temperature environment), and the amplitude of the temperature change due to the standing wave optical field from Fig. S10, T_{ac}. Using

$$\begin{aligned} A &= BT_{\mathrm{dc}} \\ B &= \frac{T_{\mathrm{ac}}\omega}{\sqrt{T_{\mathrm{dc}}^2 - T_{\mathrm{ac}}^2}} \end{aligned} \tag{S17}$$

and the values of T_{dc} and T_{ac} reported in the previous subsections, we get $A = 222425$ K W^{-1} (independent of thermal conductivity k), and $B = 1257$ ($k = 2$ W m^{-1} K^{-1}) or $B = 3971$ ($k = 20$ W m^{-1} K^{-1}).

The forcing parameters C (parametric) and D (direct) can be estimated from other simulations. By calculating the difference between eigenfrequencies of our structure at the minimum and maximum temperatures of Fig. S10, $\Delta\omega_0$ for the fundamental membrane mode, we can use $C = 2\Delta\omega_0/(\omega_0 T_{\mathrm{ac}})$. We extract $\Delta\omega = 2\pi\times 200$ Hz from a simulation where we impose only the stress change due to the thermal behavior, which gives $C \simeq 0.01$ K^{-1} The parameter D should be zero, because the tensile stress means that a temperature change does not lead to a direct out-of-plane displacement. This is true if the out-of-plane heat gradient is small enough, the tensile stress does not reach zero, and as long as our entire structure has the same thermal expansion coefficient (i.e. is from the same material). The latter is what distinguishes us from previous work on the optothermal excitation (bolometric backaction) driving a cantilever into self-oscillation [36], where the gold layer induces deflection under temperature change such that $D \neq 0$.

Finally, the we take mechanical quality factor $Q = 1\times 10^6$ and $\psi = 1$. The latter we implicitly assumed already in Sec. I D 3, where the standing wave field cancels out fully (modulated power goes to zero). This is likely an overestimation due to losses and transmission through the Si backplane. The assumptions behind the derivation of [27] leading to Eq. (S15) are somewhat different than for our system, since they use small displacement $x \ll \lambda$.

We evaluate Eq. (S15) and plot the power necessary for a limit-cycle of radius R in Fig. S11. We distinguish between the absorption that we expect for an unpatterned Si_3N_4 film (1% light absorption, blue), and if it would be enhanced a factor 3 by the standing wave (orange). For non-zero limit cycle amplitude R, the orange curve lies below the (maximum) power we send in, 3.6 mW indicated by the black dashed line, which shows that our membrane can be driven to self-oscillation by the optothermal parametric effect. This requires a slightly higher absorption than would be expected based on the material parameters, which can be achieved due to the internal standing optical waves shown in the previous section. Fig. S11 shows that limit cycles with amplitudes larger than $R \simeq 30$ nm can exist. For $R \to 0$, no limit cycle appears which is due to $D = 0$ in our system. By evaluating the second derivative of P with respect to R, $\mathrm{d}^2P/\mathrm{d}R^2$, we find that the Hopf bifurcation is supercritical and thus our limit-cycle is stable [27].

The optothermal parametric drive forms an effective periodic modulation, which could also lead to a frequency comb, similar to the dielectrophoretic force. By including a periodic variation in the frequency ω_0, we simulate this effect. The equation of motion is

$$\ddot{x} + \gamma\dot{x} + \omega_0^2(1 + F_{\mathrm{th}}\sin 2\omega_0 t)x = 0, \tag{S18}$$

such that the parametric term oscillates at twice the frequency of motion. With $F_{\mathrm{th}} = 1\cdot 10^{-5}$, we are close to the simulated frequency shift $\Delta\omega$. Numerically integrating Eq. (S18) yields an increase of velocity over time (even in the presence of damping $\gamma/2\pi = 0.2$ Hz). However, the higher harmonics

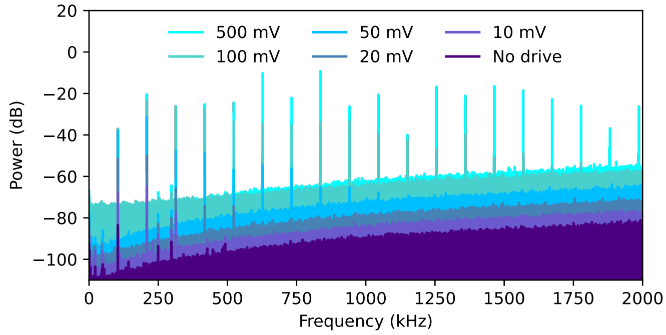

FIG. S12. **Comb with sine drive.** Observed velocity power spectra for different drive powers of a 104.524 kHz sine wave. The overtones appear when the resonator is driven with high powers on resonance, such that the displacement amplitude is large. The individual traces are offset by 5 dB vertically.

generated by the inclusion of this parametric drive are negligible, 60 dB lower than the fundamental mode. Higher harmonics (above $2\omega_0$) do not appear visibly in the spectrum. While the optothermal parametric drive is sufficiently strong to drive the membranes to self-oscillation, it is not strong enough to result in a frequency comb without the dielectrophoretic force.

To conclude this section, we have simulated the optical and thermal behavior of our resonators and analyzed the possibility of self-oscillation. We find that the motion of the membrane through the standing wave optical field modulates the absorbed light intensity, which in turn modulates the tensile stress in the material. This is sufficiently strong to drive our resonator to self-oscillation, if the absorption is slightly higher than what is expected based on purely the material parameters.

E. Overtone comb with piezo driving

In this section, we demonstrate that it is possible to generate the overtone frequency comb through inertial driving with a piezo shaker. We do this by using several membranes that do not show any sign of overtones when the laser is positioned anywhere on the membrane for at least five minutes. This way, we exclude thermal parametric driving. The piezo shaker is mounted on the back-side of the sample holder and driven via an Agilent 33220A arbitrary waveform generator and a a FLC A400 voltage amplifier with 20× gain.

In Fig. S12, we show the velocity power spectrum of a device that is driven on resonance by a sine wave of varying power. Without any drive, the fundamental mode (104.524 kHz) can be observed, together with the second and third modes above 250 kHz. At higher powers, the power in the first mode grows and overtones appear. For the highest drive powers, we retrieve a comb similar to the one reported in the main text. This demonstrates that using an external drive tone, we can utilize the overtone mechanism to generate a frequency comb.

If the comb can be generated with the piezo by applying a white noise drive, it would be possible to avoid an external frequency reference that must be matched to the device. In Fig. S13**a**, we apply a white noise drive of varying peak-to-peak amplitude. Again, the overtone comb is absent for lower drive powers, but clearly present once the drive is sufficiently strong. Fig. S13**b** shows the fundamental mode for the lowest and highest drive cases. We perform a similar analysis of the white-noise driven comb dynamics as for the optothermal parametrically driven comb in the main text. The displacement of the mechanical resonator over time as we increase the drive power is shown in Fig. S13**c**, starting from 0 V at $t \leq 2.5$ s to 3 V at $t \geq 25$ s. Compared to those obtained via thermal parametric driving, the white noise drive results in a less smooth displacement signal. The amplitude is also noticeably smaller, which may be related to using a different displacement encoder, which is not impedance-matched to the oscilloscope used to record the time signal. By extracting the different overtones, Fig. S13**d**, we see the fundamental mode increase in power first, the first and second overtone grow some seconds later and the higher overtones only appear once the amplitude has grown sufficiently. This matches the behavior of the comb shown in the main text. The bumps between $t = 20$ s and $t = 28$ s are due to the waveform generator internal switches.

We have shown that we can generate the overtone frequency comb using different driving mechanisms. Using inertial driving via a piezoelectric shaker, we observe qualitatively the same comb behavior as when using the thermal parametric drive. However, the white noise drive results in a noisier displacement signal and comb teeth powers. To highlight this, we show the displacement power spectrum around the first three tones in Fig. S13**e**, where the vertical lines indicate fast changes of comb power. This, combined with the added complexity of requiring a piezoelectric shaker, voltage source and amplifier, motivates the choice of using thermal parametric driving for the majority of the measurements in this work.

F. Extension via comb interactions

The bandwidth (span) of a frequency comb is an important property for many applications. While the overtone comb already performs on-par with the largest bandwidth mechanical combs (Table S1), we illustrate how the bandwidth can be extended further. This can be done by letting the frequency comb interact with a higher-order mechanical eigenmode ω_h of the membrane, to generate a comb with frequency spacing ω_0 centered around ω_h. This comb around ω_h has harmonics appearing on both sides of ω_h. In contrast, the comb of ω_0 has harmonics only on one side. This means that the interaction with ω_h could double the span.

We observe a copy of the fundamental mode overtone comb (ω_0 spacing) symmetrically around the higher-order mode of the system. In Fig. S14**a**, we plot the spectrum around a higher order at $\omega_h = 2083.3$ kHz which we designate as the (5,5) mode shown in the inset. The simulated frequency of the (5,5) mode is at 2046 kHz, and this is the closest mode with significant mode amplitude at the readout position (same as in Fig. 2**c** of the main text). The symmetric pattern of ω_0-

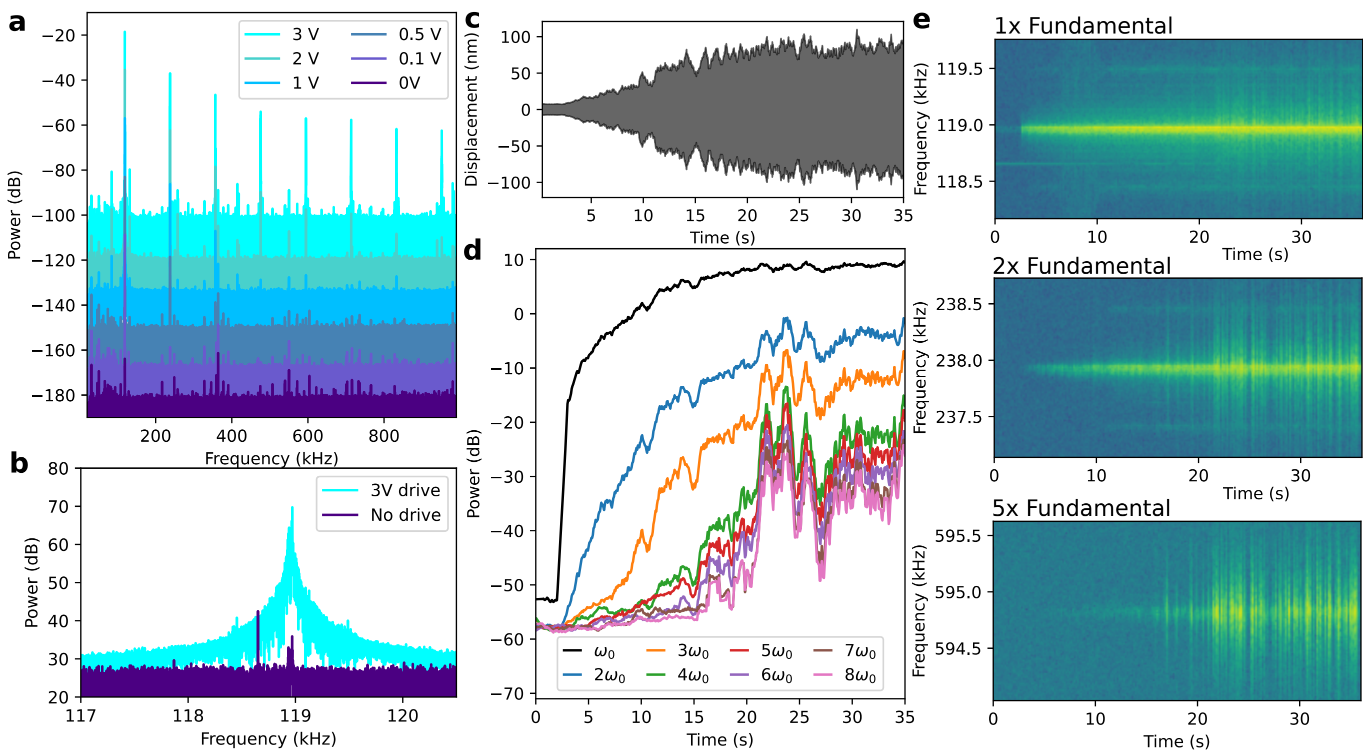

FIG. S13. **Overtone frequency comb through piezo driving.** **a:** Displacement power spectrum for various white noise driving powers. The overtone frequency comb only appears for the highest driving powers. **b:** Fundamental mechanical mode without and with driving. **c:** Displacement of the resonator as the drive power is slowly increased from 0 to 3 V. The displacement shows a less smooth signal than those obtained through thermal parametric driving in the main text. **d:** Extracted strengths of the individual comb teeth over time from the signal shown in **c**. Jumps around 25 s likely originate from the waveform generator internal switches. **e:** spectral maps of fundamental and first two overtones. The jumps observed in **d** cause the vertical lines.

spaced comb teeth is offset from the overtones of ω_0 (black dashed lines).

To corroborate this interaction between the frequency comb as a whole and a higher-order mode of the membrane, we closely study the frequency of two of the comb teeth over time. Originating from the same measurement as Fig. 3 of the main text, the fundamental mode at ω_0 has a 9 Hz upwards frequency shift over the duration of the measurement. In Fig. S14**b**, we plot the spectrum of two of the comb teeth (the first ones symmetrically around the central peak). These peaks show the same shift in frequency, but one shifts upwards while the other shifts downwards in frequency; they are clearly spaced around the central (5,5) mode peak by ω_0. The teeth further away from the center follow the $9n$ Hz scaling of the fundamental mode comb as described in the main text. This interaction thus unlocks the possibility of shifting the overtone comb upwards in frequency and thereby extend its span.

G. Effect of optics on comb and membrane

In this section, we demonstrate the overtone frequency comb on multiple devices to corroborate the measurements shown in the main text. Additionally, we also describe the visible changes in the membranes under an optical microscope when driven into the overtone comb regime, and we study the effect of the optics (power and focus) on the frequency comb.

1. *Comb prevalence and visible deformation*

We observe the overtone frequency comb behavior on multiple devices, shown in Fig. S15**a**. We fabricated two nominally identical chips containing 25 membranes each. The membranes are identical with the exception of the photonic crystal parameters, as described in earlier work [26]. All of the designs feature the same internal modes that allow for strong absorption of $\lambda = 633$ nm light as in Sec. I D, albeit at slightly different wavelengths. Each chip contains the same set of 5 photonic crystal designs, oriented as marked in Fig. S15**a**.

Of the 45 surviving devices on the two chips, 27 demonstrated the frequency comb behavior based on optothermal parametric driving (Sec. I D), and the remainder could be driven into the overtone comb regime by sufficient piezoelectric shaking (Sec. I E). This confirms the generality of the overtone mechanism.

We can corroborate the relation of the overtone comb and (powerful) heating through absorption by studying the mem-

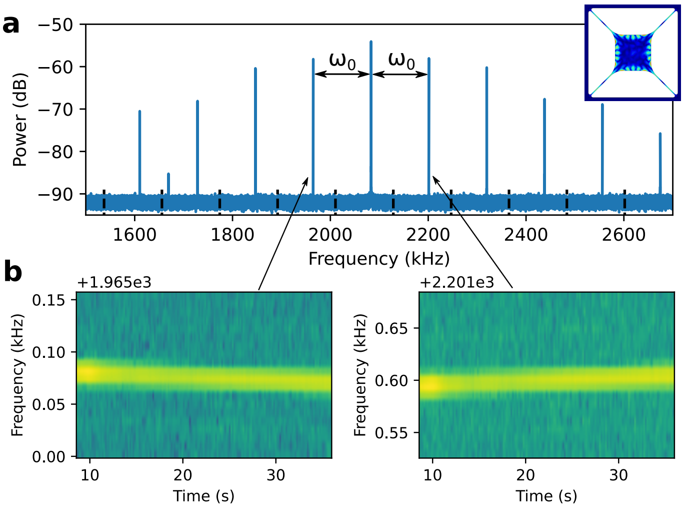

FIG. S14. **Comb interactions.** **a**: Spectrum around a higher-order mechanical mode, showing a symmetrized copy of frequency comb of the fundamental mode, ω_0. The teeth are offset from the integer multiples of the original mode (dashed lines), but still equally spaced by ω_0. Inset shows mode shape of the (5,5) mode expected to be the center peak at 2083.3 kHz. Readout position is identical to Fig. 2**c** of the main text, where the (5,5) mode has considerable amplitude. **b**: The teeth of the secondary comb follow the 9 Hz shift of the fundamental comb mode as in Fig. 3, and shift symmetrically away from the center peak.

branes using the microscope of the laser Doppler vibrometer. The presence of the optothermal parametrically driven frequency comb behavior is correlated with visible changes in the membrane structure. We show this by taking a microscope image immediately after the laser spot is moved to the center of the membrane (Fig. S15**b**, left column), and > 30 s later when the system has reached steady state (Fig. S15**b**, right column). There is an interference pattern at the edges of the membrane, which is likely from the change in photonic crystal pattern due to a stress-gradient from the edge of the Si_3N_4. The laser beam heats the membrane and via the thermal expansion of the Si_3N_4, the stress changes such that this edge deforms and the interference pattern changes.

We show three devices to illustrate different changes observable in this interference pattern in Fig. S15**b**. In some devices (top row), the pattern disappears completely, which suggests these have the strongest absorption: The deformation is larger than in other devices and the steady-state temperature is thus high. Simultaneously, these devices show strong frequency combs. In other devices (Fig. S15**b**, middle row), the interference pattern disappears only partially. This is typically along one diagonal, which suggests that the stress along the other diagonal is not fully removed; we can switch between the diagonals by positioning our laser spot. The steady-state temperature is likely less than in the case where the pattern disappears completely, and also the frequency comb appears less pronounced. Finally, a few devices show a small discoloration around the laser spot, which suggests that the interference pattern is modified only slightly. These devices display only weak frequency combs. These behaviors corroborate that the thermal parametric driving through absorption of the light is the origin of the frequency combs that we observe.

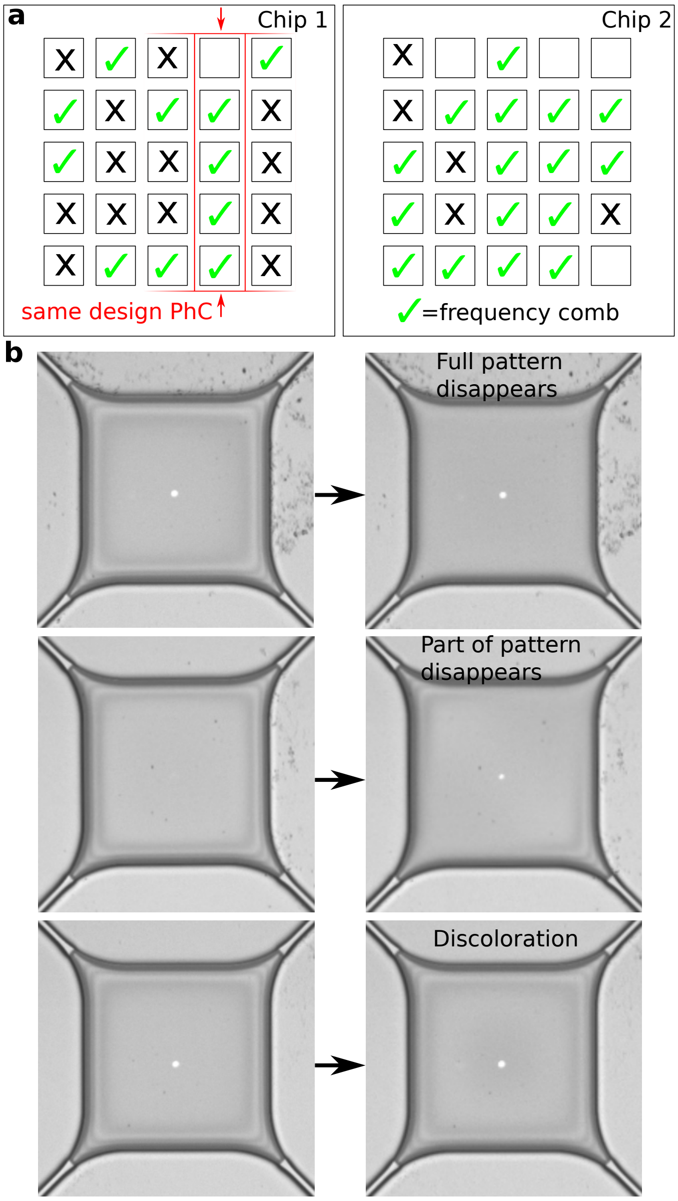

FIG. S15. **Visual changes in membrane structure. a:** Schematic sample maps of two nominally identical chips containing 25 membrane devices each, using 5 different photonic crystal designs. Green checkmarks indicate which devices show frequency comb behavior when optically addressed, blank squares show membranes collapsed during fabrication. **b**: Microscope images of three different devices before (left) and during (right) frequency comb behavior, showing visible changes. The color pattern along the outer edge of the membrane either disappears fully (top), partially (middle) or shows as a slight discoloration centered around the laser spot (bottom), indicating induced change in the photonic crystal lattice spacing and hole radius.

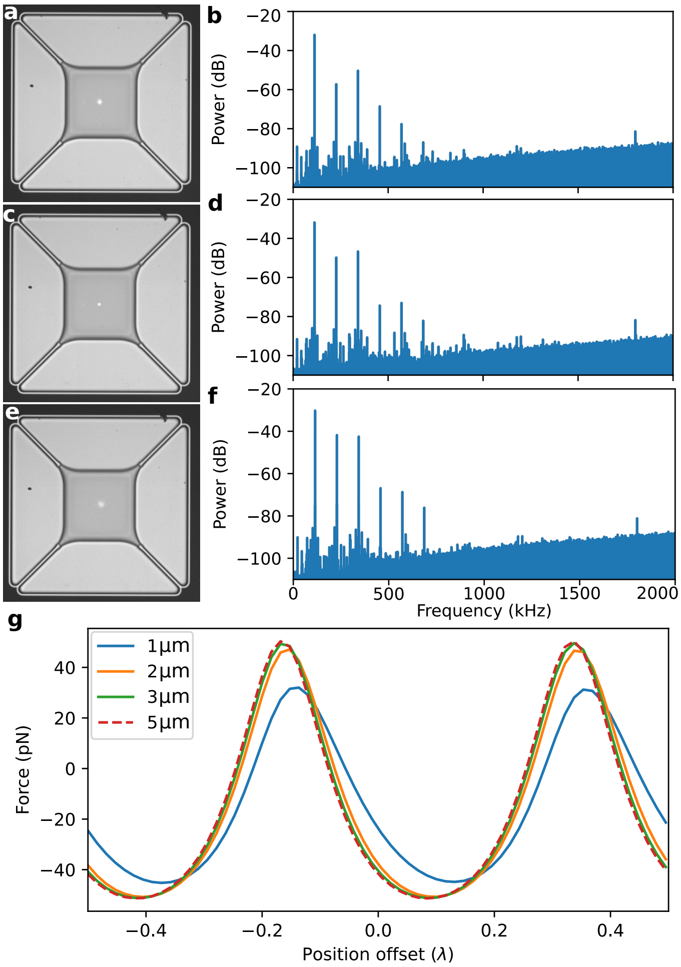

FIG. S16. **Comb dependence on laser focus. a,c,e:** Microscope images of the same device with the laser focus before (**a**), at (**c**) and after (**e**) the plane of the membrane structure. **b,d,f:** Frequency comb spectra measured with the respective laser foci, showing no major change. **g:** Simulated trapping force as a function of beam waist for constant total beam power.

2. *Comb dependence on optical focus*

We study the effect of laser focus on the frequency comb behavior. By deliberately defocusing the laser beam, and using a membrane that has a weak frequency comb behavior, we expect the focus to change the number of overtones appearing in the spectrum.

In Fig. S16**a,c,e**, we show microscope images of the laser focus before, at and after the plane of the membrane. In **b,d,f**, we plot the velocity power spectrum measured from the devices with their respective foci, which are nearly identical. This shows that the formation of the frequency comb does not depend significantly on the laser focusing. Simulations of the optical trapping force as a function of beam waist (Fig. S16**g**) confirm this behavior: The trapping force does not change for beam waists > 3 μm. Simultaneously, the thermal parametric force is limited to the area of the beam spot, so as long as the total beam power is constant, the changes in power density and beam area will cancel and the thermal parametric driving will be constant. This shows that the generation of an overtone frequency comb does not require a tightly focused laser beam.

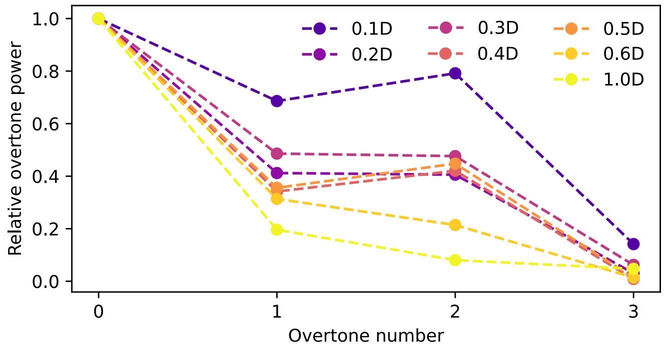

FIG. S17. **Comb dependence on optical power.** Relative powers in the individual overtones, normalized the overtone powers without filter and to the power of the fundamental mode for each filter setting. With less optical power, the overtones decrease in power with respect to the fundamental mode, as simulated in Fig. S3. The labeled optical densities correspond to a transmission of 0.1D : 79%, 0.2D : 63%, 0.3D : 50%, 0.4D : 32%, 0.5D : 32%, 0.6D : 25%, 1.0D : 10%.

3. *Comb dependence on optical power*

We also study the effect of laser power on the frequency comb. We can add an absorptive neutral density filter to reduce the power of the optical beam. This decreased F_o and should decrease the power of the overtones with respect to the fundamental mode, as simulated in Fig. S3. In Fig. S17, we show the relative overtone powers measured on a single device, for various filter strengths. The power in every overtone is normalized to the overtone power for the measurement without optical filter. Subsequently, the powers of the individual filter traces are then normalized for the power in their respective fundamental mode (overtone number 0). F_o affects the power relative to the fundamental mode, not the absolute power.

From Fig. S17, we see a trend where the higher optical density filters lead to lower relative overtone powers. This matches the simulations shown in Fig. S3. For higher density filters and higher overtone numbers ($n \geq 4$), the signal quality is too poor extract a meaningful relative overtone power. Nonetheless, it is a clear validation of the model of Eq. (S2) as the correct description of the source of the frequency combs.

H. Comb stability

An important property of a frequency comb is the stability of the comb teeth over time, both in amplitude and in frequency. To gain insight in how our overtone frequency comb performs, we park our laser on a single device and measure

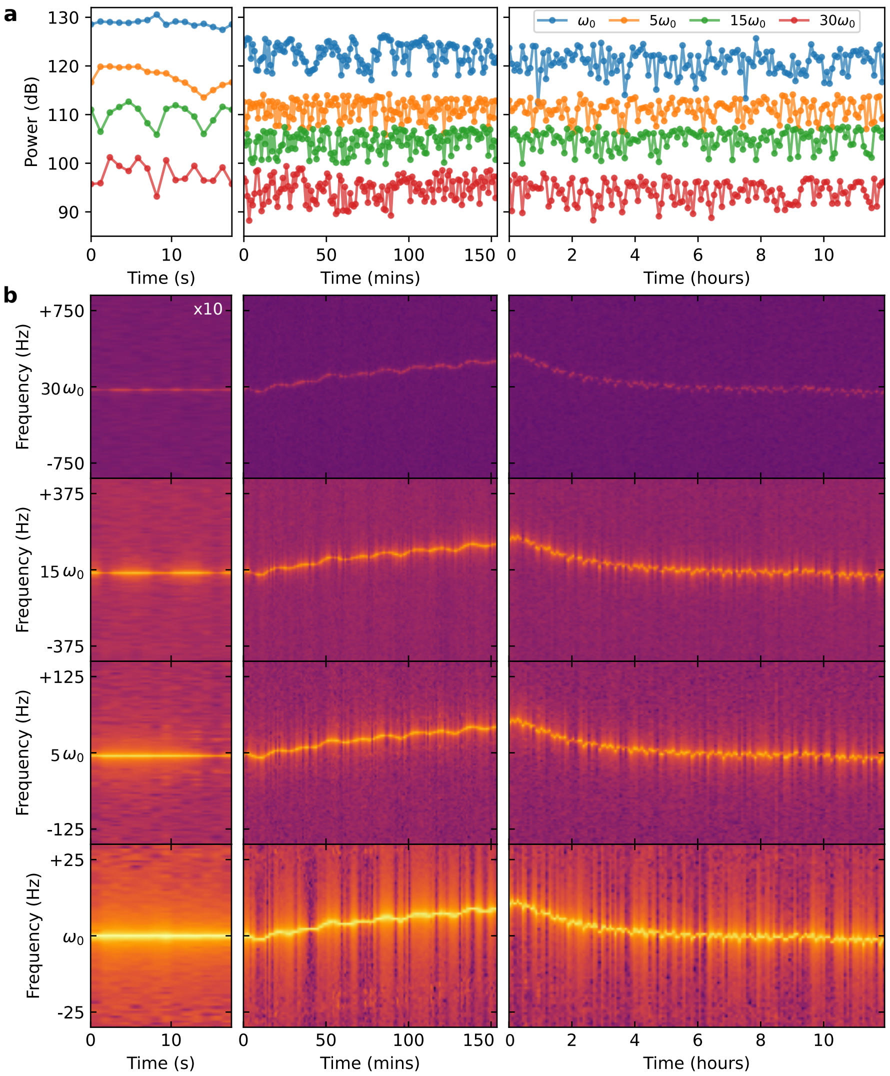

FIG. S18. **Comb stability.** **a**: Extracted power of different overtones (ω_0, $5\omega_0$, $15\omega_0$ and $30\omega_0$, measured on the same device for several timescales. There is no correlation in the amplitudes on timescales longer than a minute, but there appears to be a beating pattern on short timescales. **b:** Frequency of different overtones, measured for several timescales. The frequency is stable on short timescales, but drifts due to temperature on timescales longer than a minute. The drift is the same (scaled by overtone number) for all overtones.

the spectrum on various timescales. From each spectrum, extract the frequency and amplitude of some of the overtones, and plot them in Fig. S18.

In Fig. S18**a**, we plot the power of the overtones at ω_0, $5\omega_0$, $15\omega0$ and $30\omega_0$, offset vertically for clarity. On the timescales > 1 min, the amplitude of the individual overtones fluctuates around a constant mean with a 95% confidence interval between 2.0 dB and 2.5 dB. On shorter timescales, a correlation between the amplitudes is visible which can be reproduced by simulations if decay is included.

In Fig. S18**b**, we show the frequency of the same set of overtones, where the width of the displayed part of the spectrum is proportional to the overtone number. The color indicates power and is the same for the bottom three rows, but multiplied by a factor 10 for the top row for clarity. All comb teeth display the same frequency shift over time, scaled by overtone number. This correspondence is *exact*, to within the resolution of our measurement. On short timescales (< 16 s), the frequency of the fundamental mode is stable to within the resolution of our measurement (0.78 Hz). However, we can use the exact frequency correspondence and observe a slight shift of the overtone at $30\omega_0$, 4.68 Hz over the measurement time, or $7.9 \times 10^{-8}\,\mathrm{s}^{-1}$ relative frequency stability. This is likely limited by the environment temperature causing a frequency shift of the mechanical mode. That shift is clearly visible as the slow drift on longer timescales, as the right-most column was measured overnight and the frequency seems to stabilize. There are also oscillations on a 10 min timescale, which we attribute to the room airconditioning. If we select the most stable 6-hour period of our overnight measurement, the fractional stability reaches $7.5 \times 10^{-10}\,\mathrm{s}^{-1}$.

I. Noise in overtone frequency combs

We study fluctuations in the frequency comb as a response to (thermal) noise. By starting from Eq. 1 and including a Langevin force noise term $\xi(t)$, we obtain

$$\ddot{x} + \gamma\dot{x} + \omega_0^2 x = F_o \sin\left(\frac{4\pi}{\lambda}(x - x_{\rm off})\right) + F_{\rm d}e^{i\omega_d t} + \xi(t), \quad (S19)$$

Where the noise has zero mean $\langle\xi(t)\rangle = 0$ and is correlated $\langle\xi(t)\xi(t')\rangle = 2\gamma T\delta(t - t')$ such that we have assumed Markovian noise at temperature T, valid since resonator $Q = \frac{\omega_0}{\gamma} \gg 1$ [37]. We expand the sine-squared nonlinear term in the same way as in Sec. I A, where we keep all the terms since the displacement is not small. We can rewrite this to

$$\ddot{x} + \gamma\dot{x} + \omega_0^2 x = \sum_n^{\infty} \alpha_n x^n + F_{\rm d}e^{i\omega_d t} + \xi(t), \quad (S20)$$

such that if $x_{\rm off} = 0$, all odd coefficients (α_1, α_3, α_5,...) are zero. Let us first consider the oscillation of only the first harmonic term, $x = \frac{1}{2}\left(Ae^{i\omega_0 t} + A^* e^{-i\omega_0 t}\right)$. Then expand the series of $\alpha_n x^n$ and truncate all the tones that are not resonant. The first few terms read

$$\begin{aligned}
\alpha_1 x &= \frac{\alpha_1}{2}\left(Ae^{i\omega_d t} + A^* e^{-i\omega_d t}\right) \Rightarrow \alpha_1 x\\
\alpha_2 x &= \frac{\alpha_2}{4}\left(A^2 e^{2i\omega_d t} + 2|A|^2 + A^{*2} e^{-2i\omega_d t}\right) \Rightarrow 0\\
\alpha_3 x &= \frac{\alpha_3}{8}\left(\begin{aligned}&A^3 e^{3i\omega_d t} + 3|A|^2 A e^{i\omega_d t}\\ &+ 3|A|^2 A^* e^{-i\omega_d t} + A^{*3} e^{-3i\omega_d t}\end{aligned}\right) \Rightarrow \frac{3\alpha_3}{4}|A|^2 x\\
\alpha_4 x &= \frac{\alpha_4}{16}\left(\begin{aligned}&A^4 e^{4i\omega_d t} + 4|A|^2 A^2 e^{2i\omega_d t} + 6|A|^4\\ &+ 4|A|^2 A^{*2} e^{-2i\omega_d t} + A^{*4} e^{-4i\omega_d t}\end{aligned}\right) \Rightarrow 0.
\end{aligned} \quad (S21)$$

We can thus rewrite Eq. (S20) as

$$\ddot{x} + \gamma\dot{x} + \omega_0^2 x = \left[\alpha_1 x + 0 + \frac{3\alpha_3}{4}|A|^2 x + 0 + \frac{5\alpha_5}{8}|A|^4 x + ...\right] + F_{\rm d}e^{i\omega_d t} + \xi(t). \quad (S22)$$

Now we can introduce a new set of coefficients β_n such that $\beta_1 = 1$, $\beta_2 = 0$, $\beta_3 = \frac{3}{4}$, $\beta_4 = 0$, $\beta_5 = \frac{5}{8}$. Rearranging the terms now gives us

$$\begin{aligned}
\ddot{x} + \gamma\dot{x} + \left[1 - \sum_n \alpha_n\beta_n|A|^{n-1}\right]\omega_0^2 x &= F_{\rm d}e^{i\omega_d t} + \xi(t)\\
\ddot{x} + \gamma\dot{x} + \Omega_0^2 x &= F_{\rm d}e^{i\omega_d t} + \xi(t).
\end{aligned} \quad (S23)$$

This way, we work with the frequency of the first harmonic, Ω_0, taking into account the shift due to the non-linearity. In the case where $x_{\rm off} = 0$, all odd $\alpha_n = 0$, and all even $\beta_n = 0$ in general, which implies that the frequency of the first harmonic is not shifted by the non-linearity. Intuitively, that makes sense since the electric field is symmetric around the resonator rest position for $x_{\rm off} = 0$.

We obtain the amplitude of the first harmonic by switching off the noise $\xi(t)$, and get

$$|A|^2 = \frac{F_d^2}{\left(\left[1 - \sum_n \alpha_n\beta_n|A|^{n-1}\right]\omega_0^2 - \omega_d^2\right)^2 + \gamma^2\omega_d^2}. \quad (S24)$$

Similarly, we can find the amplitude response to fluctuations $\xi(t)$ by turning off the coherent drive,

$$\ddot{x} + \gamma\dot{x} + \Omega_0^2 x = \xi(t). \quad (S25)$$

In Fourier space, we then get

$$X(\omega) = \frac{\xi(\omega)}{\Omega_0^2 - \omega^2 + i\gamma\omega} \quad (S26)$$

with autocorrelation

$$\langle X(\omega)X(\omega')\rangle = \frac{2\gamma k_{\rm B}T\delta(\omega + \omega')}{(\Omega_0^2 - \omega^2 + i\gamma\omega)(\Omega_0^2 - \omega'^2 + i\gamma\omega')}. \quad (S27)$$

Then we can find the mean square amplitude response to fluctuations as

$$\begin{aligned}
\overline{\delta x^2} = \left\langle X(t)^2\right\rangle &= \int \frac{d\omega}{2\pi}\frac{d\omega'}{2\pi}\langle X(\omega)X(\omega')\rangle e^{i(\omega+\omega')t}\\
&= \frac{2k_{\rm B}T}{\Omega_0^2}.
\end{aligned} \quad (S28)$$

Thus the mean square amplitude of the first harmonic due to thermal fluctuations depends on the optical non-linearity via Ω_0^2.

Now to obtain the net motion we consider both driving and noise terms (use Eq. (S20)) and make the ansatz that our solution consists of a set of harmonics with a perturbation δx

$$x = \sum_{n=1}^{\infty} A_n e^{in\omega_0 t} + \sum_{n=1}^{\infty} A_n^* e^{-in\omega_0 t} + \delta x = B + \delta x. \quad (S29)$$

The nonlinear term $\sum_n \alpha_n x^n$ from Eq. (S20) can then be expanded and simplified by neglecting higher-order noise terms and keeping only the linear one. If $\delta x \ll B$, $(B + \delta x)^n \simeq B^n + nB^{n-1}\delta x$. Now if we let the sum in the nonlinear term go to a reasonable finite number $N_{\rm max}$, we split the sum and shift the index of one of the two,

$$\begin{aligned}
\sum_{n=1}^{\infty} \alpha_n(B^n + nB^{n-1}\delta x) &= \sum_{n=1}^{N_{\rm max}} \alpha_n B^n + \sum_{n=0}^{N_{\rm max}-1} \alpha_{n+1}(n+1)B^n\delta x\\
&= \alpha_1\delta x + \sum_{n=1}^{N_{\rm max}-1} B^n(\alpha_n + \alpha_{n+1}(n+1)\delta x) + \alpha_{N_{\rm max}} B^{N_{\rm max}}.
\end{aligned} \quad (S30)$$

If we then have $\alpha_n \simeq \alpha_{n+1}$ for all reasonable n, and small noise such that $\delta(n + 1) \ll 1$, we can then absorb the term at $N_{\rm max}$ back into the sum,

$$\sum_{n=1}^{\infty} \alpha_n(B^n + nB^{n-1}\delta x) \simeq \alpha_1\delta x + \sum_{n=1}^{N_{\rm max}} \alpha_n B^n. \quad (S31)$$

Thus we obtain the same solution in terms of the harmonics as we would do without considering fluctuations δx, with only the additional term $\alpha_1\delta x$. The equation of motion thus becomes

$$\sum_{n=1}^{\infty} A_n \left(\omega_0^2 - n^2\omega_d + in\gamma\omega_d\right) e^{in\omega_d t} + \ddot{\delta x} + \gamma\dot{\delta x} + \omega_0^2 \delta x = \alpha_1 \delta x + \sum_{n=1}^{N_{\max}} \alpha_n B^n + F_d e^{i\omega_d t} + \xi(t) \tag{S32}$$

Based on the previous analysis, we can linearize this to

$$\sum_{n=1}^{\infty} A_n \left(\omega_0^2 - n^2\omega_d + in\gamma\omega_d\right) e^{in\omega_d t} + \ddot{\delta x} + \gamma\dot{\delta x} + \Omega_0^2 \delta x + F_d e^{i\omega_d t} + \xi(t) \tag{S33}$$

where $\Omega_m^2 = \left[1 - \sum_n \alpha_n \beta_n |A|^{n-1}\right] \omega_0^2$. While the amplitudes A_n are obtained in Sec. I A, this analysis shows that the root-mean square fluctuations stay constant at $\frac{2k_B T}{\Omega_m^2}$ for all values of n. This indicates that the comb generation does not cause additional fluctuations or noise.

Thus we have confirmed our observations of Fig. 3**f** in the main text: The Lorentzian linewidth of our resonator fundamental mode is the same in the thermal regime as it is when driven into the overtone comb, since the optical non-linearity does not add noise to the system.

J. Phase-coherence

To show the phase-coherence of all tones within the comb, we take a closer look at the time domain signal. When the overtones at $2, 3, 4, ... \times \omega_0$ have considerable amplitude, we can extract the phases of each component separately from the shape of a single period in the time domain. To do so, fit the sum of the first n cosine terms with amplitude coefficients $a_1, ..., a_n$ and phase offsets $\phi_1, ..., \phi_n$ using

$$A(t) = a_1 \cos(2\pi\omega_0 t + \phi_1) + a_2 \cos(4\pi\omega_0 t + \phi_2) + ... + a_{16} \cos(32\pi\omega_0 t + \phi_n). \tag{S34}$$

When all phase offsets are the same, $\phi_1 = \phi_2 = ... = \phi_n$, the overtone comb is phase-coherent.

To fit Eq.(S34), we extract the amplitudes of the first $n = 16$ overtones from the measurement of the whole time signal ($\sim$ 36 s). This we use as an initial guess for a fit that optimizes them to the final amplitudes that best describe a single period ($\sim$ 8 µs). We calculate two curves, one where all phase offsets $\phi_1, ..., \phi_{16}$ are the same (orange in Fig. S19**a,b**) and one where all the phase offsets are random (black). From this, it is clear that all overtones have the same phase offet and thus the overtone comb is phase-coherent.

[1] L. Q. Zhou, G. Colston, M. J. Pearce, R. G. Prince, M. Myronov, D. R. Leadley, O. Trushkevych, and R. S. Edwards, Applied Physics Letters **111**, 011904 (2017).
[2] F. Yang, F. Rochau, J. S. Huber, A. Brieussel, G. Rastelli, E. M. Weig, and E. Scheer, Physical Review Letters **122**, 154301 (2019).
[3] F. Yang, F. Hellbach, F. Rochau, W. Belzig, E. M. Weig, G. Rastelli, and E. Scheer, Physical Review Letters **127**, 014304 (2021).
[4] J. S. Ochs, D. K. J. Boneß, G. Rastelli, M. Seitner, W. Belzig, M. I. Dykman, and E. M. Weig, Physical Review X **152**, 041019 (2022).
[5] Q. Yang, L. Xu, R. Huan, Z. Jiang, A. Ganesan, and X. Wei, in *2021 IEEE 16th International Conference on Nano/Micro Engineered and Molecular Systems* (2021).
[6] I. Mahboob, Q. Wilmart, K. Nishiguchi, A. Fujiwara, and H. Yamaguchi, Applied Physics Letters **100**, 113109 (2012).
[7] I. Mahboob, R. Dupuy, K. Nishiguchi, A. Fujiwara, and H. Yamaguchi, Applied Physics Letters **109**, 073101 (2016).
[8] M. J. Seitner, M. Abdi, A. Ridolfo, M. J. Hartmann, and E. M. Weig, Physical Review Letters **118**, 254301 (2017).
[9] A. Ganesan, C. Do, and A. Seshia, Physical Review Letters **118**, 033903 (2017).
[10] A. Ganesan, C. Do, and A. Seshia, Applied Physics Letters **100**, 021906 (2018).
[11] D. A. Czaplewski, C. Chen, D. Lopez, O. Shoshani, A. M. Eriksson, S. Strachan, and S. M. Shaw, Physical Review Letters **121**, 244302 (2018).
[12] X. Wang, Q. Yang, R. Huan, Z. Shi, W. Zhu, Z. Jiang, Z. Deng, and X. Wei, Applied Physics Letters **120**, 173506 (2022).
[13] M. Goryachev, S. Galliou, and M. E. Tobar, Physical Review Research **2**, 023035 (2020).
[14] A. Chiout, F. Correia, M.-Q. Zhao, A. T. C. Johnson, D. Pierucci, F. Oehler, A. Ouerghi, and J. Chaste, Applied Physics Letters **119**, 173102 (2021).
[15] A. Keşkekler, H. Arjmandi, P. G. Steeneken, and F. Alijani, Nano Letters **22**, 6048 (2022).
[16] G. Siegmund, in *Eighth international conference on vibration measurements by laser techniques: Advances and applications* (2008).
[17] L. Yarovoi and G. Siegmund, Measurement Science and Technology **15**, 2150 (2004).
[18] O. Dussarrat, D. Clark, and T. Moir, in *Third international conference on vibration measurements by laser techniques: Advances and applications* (1998).
[19] H. Sumali and M. Allen, in *Eighth international conference on vibration measurements by laser techniques: Advances and applications* (2008).
[20] A. Ashkin, Physical Review Letters **24**, 156 (1970).
[21] A. Ashkin, J. M. Dziedzic, J. E. Bjorkholm, and S. Chu, Optics Letters **11**, 288 (1986).
[22] H. A. Pohl, Journal of Applied Physics **22**, 869 (1951).
[23] P. Zemánek, A. Jonáš, L. Šrámek, and M. Liška, Optics Letters **24**, 1448 (1999).
[24] M.-A. Miri, G. D'Aguanno, and A. Alù, New Journal of Physics **20**, 043013 (2018).

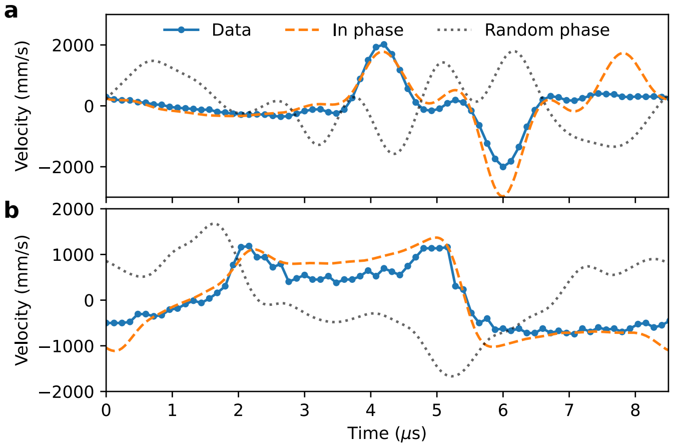

FIG. S19. **Phase coherence of overtone comb. a,b**: Single period (blue) of the measured time signals of Fig. 4**c**, together with fits containing the sum of the first 16 cosines (overtones, at ω_0, ..., $16\omega_0$). In orange, the cosines all have the same phase offset, while in black the phase offset is random. This shows the phase-coherence of our overtone comb.

[25] M. H. J. de Jong, J. Li, C. Gärtner, R. A. Norte, and S. Gröblacher, Optica **9**, 170 (2022).

[26] M. H. J. de Jong, M. A. ten Wolde, A. Cupertino, S. Gröblacher, P. G. Steeneken, and R. A. Norte, Applied Physics Letters **121**, 032201 (2022).

[27] K. Aubin, M. Zalalutdinov, T. Alan, R. B. Reichenbach, R. Rand, A. Zehnder, J. Parpia, and H. Craighead, Journal of Microelectromechanical systems **13**, 1018 (2004).

[28] J. Steinlechner, C. Krüger, I. W. Martin, A. Bell, J. Hough, H. Kaufer, S. Rowan, R. Schnabel, and S. Steinlechner, Physical Review D **96**, 022007 (2017).

[29] A. Frigg, A. Boes, G. Ren, I. Abdo, D.-Y. Choi, S. Gees, and A. Mitchell, Optics Express **27**, 37795 (2019).

[30] A. Gorin, A. Jaouad, E. Grondin, V. Aimez, and P. Charette, Optics Express **16**, 13509 (2008).

[31] M.-H. Chien, M. Brameshuber, B. K. Rossboth, G. J. Schütz, and S. Schmid, Proceedings of the National Academy of Sciences **115**, 11150 (2018).

[32] C. Gärtner, J. P. Moura, W. Haaxman, R. A. Norte, and S. Gröblacher, Nano Letters **18**, 7171 (2018).

[33] C. Zhang, M. Giroux, T. A. Nour, and R. St-Gelais, Physical Review Applied **14**, 024072 (2020).

[34] M. T. Alam, M. P. Manoharan, M. A. Haque, C. Muratore, and A. Voevodin, Journal of Micromechanics and Microengineering **22**, 045001 (2012).

[35] T. Larsen, S. Schmid, L. G. Villanueva, and A. Boisen, ACS Nano **7**, 6188 (2013).

[36] C. Metzger, M. Ludwig, C. Neuenhahn, A. Ortlieb, I. Favero, K. Karrai, and F. Marquardt, Physical Review Letters **101**, 133903 (2008).

[37] R. Benguria and M. Kac, Physical Review Letters **46**, 1 (1981).
